# Supplementary material for: The Evolution and Characterization of the RNA Interference Pathways in Lophotrochozoa
Source: Genome Biol Evol. 2024 May 7;16(5):evae098. doi: 10.1093/gbe/evae098 (PMC11114477; doi:10.1093/gbe/evae098)
Supplement: evae098_Supplementary_Data [file evae098_supplementary_data.zip › Supplementary_materials.zip!/selected_miRNAs/NoGe_selected_miRNAs/mirdeep_pdf_files/NMRB01000015.1_Notospermus_geniculatus_strain_Ushimado_scaffold15,_whole_genome_shotgun_sequence_640.pdf]

Provisional ID	:	NMRB01000015.1_Notospermus_geniculatus_strain_Ushimado_scaffold15,_whole_genome_shotgun_sequence_640
Score total	:	3690891.4
Score for star read(s)	:	3.9
Score for read counts	:	3690882.7
Score for mfe	:	1.8
Score for randfold	:	0
Score for cons. seed	:	3
Total read count	:	7239519
Mature read count	:	7239351
Loop read count	:	0
Star read count	:	168

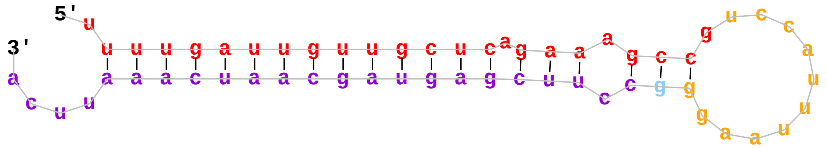

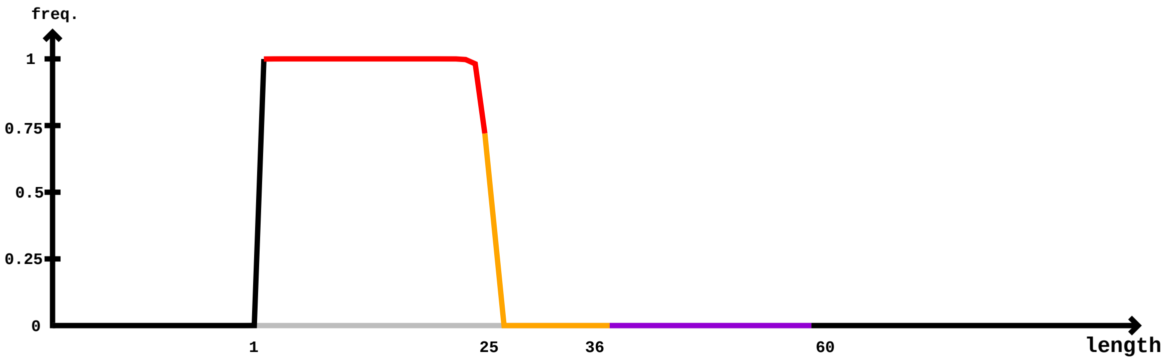


```
                     Mature                             Star
5'- caaagagucguuuguccgugguuuugauuguugcucagaaagccguccauuuaagggccuucgaguagcaaucaaauucagaggggaacgacgcuucaacauaccugauuac   -3'   obs
    caaagagucguuuguccguguuuugauuguugcucagaaagccguccauuuaagggccuucgaguagcaaucaaauucagaggggaacgacgcuucaacauaccugauuac         exp
    ..(((.(((((((.((..((.((((((((((((((.(((.(((..........))).))))))))))))))))).))..))..)).))))))).)))................. reads   mm   sample
    ..................uCuuuugauuguugcucagaaagcc.................................................................... 7     1    Mm2
    ...................guuuugauuguugGucagaaag...................................................................... 1     1    Mm2
    ...................guuuugauuguugcucGgaaag...................................................................... 1     1    Mm2
    ...................guuuugauuguugcucagaaag...................................................................... 1     0    Mm2
    ...................Cuuuugauuguugcucagaaagc..................................................................... 2     1    Mm2
    ...................guuuugauuguugcucagaaagc..................................................................... 1     0    Mm2
    ...................guuuugauuguugcucagaaagcc.................................................................... 2     0    Mm2
    ...................Cuuuugauuguugcucagaaagcc.................................................................... 67    1    Mm2
    ...................guuuugauuguugcucagaaagccU................................................................... 6     1    Mm2
    ...................Cuuuugauuguugcucagaaagccg................................................................... 9     1    Mm2
    ....................uuuugauuguugcucagU......................................................................... 3     1    Mm2
    ....................uuuugauuguugcucaga......................................................................... 3     0    Mm2
    ....................uuuugauuguugcucagaa........................................................................ 23    0    Mm2
    ....................uuuugauuguugcucagGaa....................................................................... 1     1    Mm2
    ....................uuuugauuguugcucagaaU....................................................................... 26    1    Mm2
    ....................uuuugauuguGgcucagaaa....................................................................... 1     1    Mm2
    ....................uuuugauuguugcucagaaG....................................................................... 1     1    Mm2
    ....................uuuugauuguugcucagaaa....................................................................... 137   0    Mm2
    ....................uuuugauuguugcucaCaaa....................................................................... 1     1    Mm2
    ....................uuuugauuguGgcucagaaag...................................................................... 1     1    Mm2
    ....................uuuugauuguugcucagGaag...................................................................... 8     1    Mm2
    ....................uuuuAauuguugcucagaaag...................................................................... 1     1    Mm2
    ....................uuuugauuguugcucagaaCg...................................................................... 1     1    Mm2
    ....................uuuugauuguugcucagaaag...................................................................... 1712  0    Mm2
    ....................uuuugauuguugcuUagaaag...................................................................... 2     1    Mm2
    ....................uuuugauuguugcucagaaaA...................................................................... 10    1    Mm2
    ....................uuuugauuguCgcucagaaag...................................................................... 1     1    Mm2
    ....................uuuugauuguugcucagCaag...................................................................... 8     1    Mm2
    ....................uuuugUuuguugcucagaaag...................................................................... 2     1    Mm2
    ....................uuuugauCguugcucagaaag...................................................................... 1     1    Mm2
    ....................uuuugauuguugcucaUaaag...................................................................... 7     1    Mm2
    ....................uuuugauuguugUucagaaag...................................................................... 1     1    Mm2
    ....................uuGugauuguugcucagaaag...................................................................... 1     1    Mm2
```

Mature Star

```
caaagagucguuuguccguguuuugauuguugcucagaaagccguccauuuaagggccuucgaguagcaaucaaauucagaggggaacgacgcuucaacauaccugauuac
....................uuuugauuguugcucagaaaU......................................................................   11     1    Mm2
....................uuuugaAuguugcucagaaag......................................................................   1      1    Mm2
....................Cuuugauuguugcucagaaag......................................................................   2      1    Mm2
....................uuuugauugCugcucagaaag......................................................................   1      1    Mm2
....................uuuugauuguugcucagaaGg......................................................................   1      1    Mm2
....................uuuugauuguugcucaAaaag......................................................................   2      1    Mm2
....................uuuugauuguugcucagaGag......................................................................   1      1    Mm2
....................uuuugauuguugcucGgaaag......................................................................   1      1    Mm2
....................uuuugauuguugcucagUaag......................................................................   6      1    Mm2
....................Nuuugauuguugcucagaaag......................................................................   2      1    Mm2
....................uuuugauuguugcucaCaaag......................................................................   3      1    Mm2
....................Auuugauuguugcucagaaag......................................................................   1      1    Mm2
....................uuuugauuguAgcucagaaag......................................................................   5      1    Mm2
....................uuAugauuguugcucagaaag......................................................................   1      1    Mm2
....................uuuugauuguugcucagGaagc.....................................................................   25     1    Mm2
....................uuuuUauuguugcucagaaagc.....................................................................   1      1    Mm2
....................uuuugauuguugcucagaUagc.....................................................................   1      1    Mm2
....................uuuugauuguugcucagaaagc.....................................................................   8638   0    Mm2
....................uuuugauuguCgcucagaaagc.....................................................................   10     1    Mm2
....................uuuugauuguuUcucagaaagc.....................................................................   5      1    Mm2
....................Cuuugauuguugcucagaaagc.....................................................................   1      1    Mm2
....................uuuugauuguugcucagaGagc.....................................................................   1      1    Mm2
....................uCuugauuguugcucagaaagc.....................................................................   1      1    Mm2
....................uuuugauuguugcuUagaaagc.....................................................................   3      1    Mm2
....................uuuugauuguugcGcagaaagc.....................................................................   1      1    Mm2
....................uuuugauuguugcucagCaagc.....................................................................   35     1    Mm2
....................uuuugUuuguugcucagaaagc.....................................................................   2      1    Mm2
....................uGuugauuguugcucagaaagc.....................................................................   2      1    Mm2
....................uuuAgauuguugcucagaaagc.....................................................................   3      1    Mm2
....................uuuugauuguugcucagUaagc.....................................................................   24     1    Mm2
....................uuCugauuguugcucagaaagc.....................................................................   1      1    Mm2
....................uuuuAauuguugcucagaaagc.....................................................................   1      1    Mm2
....................uuAugauuguugcucagaaagc.....................................................................   3      1    Mm2
....................uuuugauuguGgcucagaaagc.....................................................................   12     1    Mm2
....................uuuugGuuguugcucagaaagc.....................................................................   1      1    Mm2
....................uuuugauuguugcAcagaaagc.....................................................................   1      1    Mm2
....................uuuugauuguugcucagaaaUc.....................................................................   2      1    Mm2
....................uAuugauuguugcucagaaagc.....................................................................   3      1    Mm2
....................uuuugauGguugcucagaaagc.....................................................................   1      1    Mm2
....................uuuugauuguugcucaCaaagc.....................................................................   27     1    Mm2
....................uuuugauuguuCcucagaaagc.....................................................................   2      1    Mm2
....................uuuugauuguugcucagaaagA.....................................................................   23     1    Mm2
....................uuuugauuguugcCcagaaagc.....................................................................   4      1    Mm2
....................uuuugauuguugUucagaaagc.....................................................................   2      1    Mm2
....................uuuugNuuguugcucagaaagc.....................................................................   1      1    Mm2
....................uuuugauuguugcucagaaGgc.....................................................................   2      1    Mm2
....................uuuugauuguugcucagaaagU.....................................................................   79     1    Mm2
....................uuuugauAguugcucagaaagc.....................................................................   1      1    Mm2
....................uuuugauuguugcucaUaaagc.....................................................................   15     1    Mm2
....................uuuCgauuguugcucagaaagc.....................................................................   2      1    Mm2
....................Nuuugauuguugcucagaaagc.....................................................................   3      1    Mm2
....................uuuugauuguugcucaAaaagc.....................................................................   18     1    Mm2
....................uuuugauuguugcucagaaUgc.....................................................................   2      1    Mm2
....................uuuugauuguAgcucagaaagc.....................................................................   21     1    Mm2
....................uuuugauuAuugcucagaaagc.....................................................................   1      1    Mm2
....................uuuugauugCugcucagaaagc.....................................................................   1      1    Mm2
....................uuuugaAuguugcucagaaagc.....................................................................   1      1    Mm2
....................uuuugauuguugAucagaaagc.....................................................................   1      1    Mm2
....................uuuugauuguugcucUgaaagc.....................................................................   1      1    Mm2
....................uuuugauuguugcucagaaaCcc....................................................................   14     1    Mm2
....................uuuugaNuguugcucagaaagcc....................................................................   8      1    Mm2
....................uuuugauuguugcucagaaagAc....................................................................   6      1    Mm2
....................uuuugauuguugcucagUaagcc....................................................................   374    1    Mm2
....................uuuuCauuguugcucagaaagcc....................................................................   6      1    Mm2
....................uCuugauuguugcucagaaagcc....................................................................   27     1    Mm2
....................uNuugauuguugcucagaaagcc....................................................................   2      1    Mm2
....................uuuugauuguugcucGgaaagcc....................................................................   17     1    Mm2
....................uuuugauuguugcucUgaaagcc....................................................................   10     1    Mm2
....................uuuugauuguugcCcagaaagcc....................................................................   15     1    Mm2
....................uuuugauuguugAucagaaagcc....................................................................   13     1    Mm2
```

Mature **Star**

```
caaagagucguuuguccguguuuugauuguugcucagaaagccguccauuuaagggccuucgaguagcaaucaaauucagaggggaacgacgcuucaacauaccugauuac
....................uuuugauuguugcucagaaaAcc....................................................................  11      1  Mm2
....................uuuugauuguugcucagaaagGc....................................................................  3       1  Mm2
....................uuuAgauuguugcucagaaagcc....................................................................  24      1  Mm2
....................uuuugGuuguugcucagaaagcc....................................................................  16      1  Mm2
....................uuuGgauuguugcucagaaagcc....................................................................  5       1  Mm2
....................uuuugauuguugcucagaaagcG....................................................................  11      1  Mm2
....................uuuugauuguugcucaUaaagcc....................................................................  253     1  Mm2
....................uGuugauuguugcucagaaagcc....................................................................  12      1  Mm2
....................uuuugauuguugcGcagaaagcc....................................................................  6       1  Mm2
....................uuuugauuUuugcucagaaagcc....................................................................  28      1  Mm2
....................uuGugauuguugcucagaaagcc....................................................................  66      1  Mm2
....................uuuugauuguGgcucagaaagcc....................................................................  132     1  Mm2
....................uuuugauuguugcucagCaagcc....................................................................  459     1  Mm2
....................uuuugauugGugcucagaaagcc....................................................................  1       1  Mm2
....................uuuugauuguuCcucagaaagcc....................................................................  5       1  Mm2
....................uuuugauuguugcucagaaagcA....................................................................  141     1  Mm2
....................uuuugauuAuugcucagaaagcc....................................................................  11      1  Mm2
....................uuuugauuCuugcucagaaagcc....................................................................  6       1  Mm2
....................uuuugauuguugcucCgaaagcc....................................................................  2       1  Mm2
....................uuuugauuguugcucagaaaUcc....................................................................  22      1  Mm2
....................uuuugUuuguugcucagaaagcc....................................................................  47      1  Mm2
....................uuuugauuguAgcucagaaagcc....................................................................  275     1  Mm2
....................uuuugauCguugcucagaaagcc....................................................................  16      1  Mm2
....................uuuugauuguugcucaCaaagcc....................................................................  344     1  Mm2
....................Guuugauuguugcucagaaagcc....................................................................  6       1  Mm2
....................uuuCgauuguugcucagaaagcc....................................................................  25      1  Mm2
....................uuuugauAguugcucagaaagcc....................................................................  12      1  Mm2
....................uuuugauuguugcuGagaaagcc....................................................................  4       1  Mm2
....................uuuugauuguuAcucagaaagcc....................................................................  10      1  Mm2
....................uAuugauuguugcucagaaagcc....................................................................  35      1  Mm2
....................uuuugauuguCgcucagaaagcc....................................................................  142     1  Mm2
....................uuuugauuguugcuUagaaagcc....................................................................  36      1  Mm2
....................uuuugauuguuUcucagaaagcc....................................................................  32      1  Mm2
....................uuuugauuguugcucagaCagcc....................................................................  2       1  Mm2
....................uuuugauuguugcucagaUagcc....................................................................  4       1  Mm2
....................uuuugauuguugcAcagaaagcc....................................................................  21      1  Mm2
....................uuuugauuguugcucagaaagUc....................................................................  14      1  Mm2
....................uuuugNuuguugcucagaaagcc....................................................................  10      1  Mm2
....................uuuugauGguugcucagaaagcc....................................................................  6       1  Mm2
....................uuuugauugAugcucagaaagcc....................................................................  7       1  Mm2
....................uuuugauuguugcuAagaaagcc....................................................................  6       1  Mm2
....................uuuuAauuguugcucagaaagcc....................................................................  11      1  Mm2
....................uuuugaAuguugcucagaaagcc....................................................................  10      1  Mm2
....................uuAugauuguugcucagaaagcc....................................................................  22      1  Mm2
....................uuuugauuguugcucagGaagcc....................................................................  346     1  Mm2
....................Cuuugauuguugcucagaaagcc....................................................................  55      1  Mm2
....................uuuugauugCugcucagaaagcc....................................................................  10      1  Mm2
....................uuCugauuguugcucagaaagcc....................................................................  26      1  Mm2
....................uuuugCuuguugcucagaaagcc....................................................................  9       1  Mm2
....................Nuuugauuguugcucagaaagcc....................................................................  53      1  Mm2
....................Auuugauuguugcucagaaagcc....................................................................  12      1  Mm2
....................uuuugauuguugcucagaGagcc....................................................................  15      1  Mm2
....................uuuugauuguugcucagaaagcc....................................................................  124138  0  Mm2
....................uuNugauuguugcucagaaagcc....................................................................  1       1  Mm2
....................uuuugauuguugcucagaaGgcc....................................................................  82      1  Mm2
....................uuuugauuguugGucagaaagcc....................................................................  2       1  Mm2
....................uuuugauuguugUucagaaagcc....................................................................  23      1  Mm2
....................uuuugauuguugcucagaaCgcc....................................................................  5       1  Mm2
....................uuuugaCuguugcucagaaagcc....................................................................  12      1  Mm2
....................uuuugauuguugcucagaaagcU....................................................................  454     1  Mm2
....................uuuugauuguugcucaAaaagcc....................................................................  235     1  Mm2
....................uuuuUauuguugcucagaaagcc....................................................................  7       1  Mm2
....................uuuugauuguugcucagaaUgcc....................................................................  4       1  Mm2
....................uuuugaGuguugcucagaaagcc....................................................................  6       1  Mm2
....................uuuugauuguugcucagaaagUcg...................................................................  4       1  Mm2
....................uuuuAauuguugcucagaaagccg...................................................................  1       1  Mm2
....................uuuugauuguugcucaUaaagccg...................................................................  16      1  Mm2
....................uGuugauuguugcucagaaagccg...................................................................  2       1  Mm2
....................uuuugGuuguugcucagaaagccg...................................................................  2       1  Mm2
....................uuAugauuguugcucagaaagccg...................................................................  1       1  Mm2
```

Mature **Star**

```
caaagagucguuuguccguguuuugauuguugcucagaaagccguccauuuaagggccuucgaguagcaaucaaauucagaggggaacgacgcuucaacauaccugauuac
....................uuuugauuAuugcucagaaagccg...................................................................  1       1   Mm2
....................uAuugauuguugcucagaaagccg...................................................................  4       1   Mm2
....................uuuugauuguAgcucagaaagccg...................................................................  42      1   Mm2
....................uCuugauuguugcucagaaagccg...................................................................  5       1   Mm2
....................uuuugauCguugcucagaaagccg...................................................................  2       1   Mm2
....................uuuugaCuguugcucagaaagccg...................................................................  1       1   Mm2
....................Nuuugauuguugcucagaaagccg...................................................................  4       1   Mm2
....................uuuugCuuguugcucagaaagccg...................................................................  1       1   Mm2
....................uuuugauuguugcucagaaagccg...................................................................  13091   0   Mm2
....................uuuugauuguugcucagaaagccU...................................................................  289428  1   Mm2
....................uuuugNuuguugcucagaaagccg...................................................................  1       1   Mm2
....................uuuugauuguugcGcagaaagccg...................................................................  1       1   Mm2
....................uuCugauuguugcucagaaagccg...................................................................  1       1   Mm2
....................uuuugauuguuAcucagaaagccg...................................................................  1       1   Mm2
....................uuuuUauuguugcucagaaagccg...................................................................  2       1   Mm2
....................uuuugauuguuUcucagaaagccg...................................................................  3       1   Mm2
....................uuuugauuguugcucaAaaagccg...................................................................  20      1   Mm2
....................uuuugauuguugcucagaUagccg...................................................................  1       1   Mm2
....................uuuugauuguugUucagaaagccg...................................................................  1       1   Mm2
....................uuuugauuguCgcucagaaagccg...................................................................  13      1   Mm2
....................uuuugauuguugcucagaaaAccg...................................................................  3       1   Mm2
....................uuuugauuguugcucagaaagccA...................................................................  1450    1   Mm2
....................uuuugauuguugAucagaaagccg...................................................................  1       1   Mm2
....................uuuugauuguugcucUgaaagccg...................................................................  1       1   Mm2
....................Auuugauuguugcucagaaagccg...................................................................  3       1   Mm2
....................Guuugauuguugcucagaaagccg...................................................................  2       1   Mm2
....................uuuugauuguugcucagCaagccg...................................................................  70      1   Mm2
....................uuuugauuguuCcucagaaagccg...................................................................  1       1   Mm2
....................uuuugaAuguugcucagaaagccg...................................................................  1       1   Mm2
....................uuuGgauuguugcucagaaagccg...................................................................  1       1   Mm2
....................uuuugauuguugcuUagaaagccg...................................................................  6       1   Mm2
....................uuuugauuguugcAcagaaagccg...................................................................  7       1   Mm2
....................uuuugauuguugcucagaaGgccg...................................................................  8       1   Mm2
....................uuuugauAguugcucagaaagccg...................................................................  1       1   Mm2
....................uuuugauuguugcucagUaagccg...................................................................  53      1   Mm2
....................uuuugauuguugcucagaaagcUg...................................................................  4       1   Mm2
....................uuuugauuCuugcucagaaagccg...................................................................  1       1   Mm2
....................uuuuCauuguugcucagaaagccg...................................................................  1       1   Mm2
....................uuuugauuguugcucaCaaagccg...................................................................  38      1   Mm2
....................Cuuugauuguugcucagaaagccg...................................................................  5       1   Mm2
....................uuuugauuguugcucagaaagccC...................................................................  305     1   Mm2
....................uuuugUuuguugcucagaaagccg...................................................................  6       1   Mm2
....................uuuugauuguugcCcagaaagccg...................................................................  4       1   Mm2
....................uuuugauuguugcucagaaaUccg...................................................................  1       1   Mm2
....................uuGugauuguugcucagaaagccg...................................................................  5       1   Mm2
....................uuuugauuguugcucagaaagAcg...................................................................  1       1   Mm2
....................uuuugauugCugcucagaaagccg...................................................................  2       1   Mm2
....................uuuugauuguugcucagaGagccg...................................................................  2       1   Mm2
....................uuuugaGuguugcucagaaagccg...................................................................  2       1   Mm2
....................uuuugauuUuugcucagaaagccg...................................................................  1       1   Mm2
....................uuuugauuguGgcucagaaagccg...................................................................  17      1   Mm2
....................uuuugauuguugcucagaaagcGg...................................................................  1       1   Mm2
....................uuuugauuguugcucagGaagccg...................................................................  29      1   Mm2
....................uuuugauuguugcucagaaagccCu..................................................................  5       1   Mm2
....................uuuugauuguugcucagaaagccAu..................................................................  10      1   Mm2
....................uuuugauuguugcucagaaagccUu..................................................................  442     1   Mm2
.....................uuugauuguugcucagaaagc.....................................................................  3       0   Mm2
.....................uuugauuguuUcucagaaagc.....................................................................  1       1   Mm2
.....................uuugaAuguugcucagaaagcc....................................................................  1       1   Mm2
.....................uuugauuguugcucagaaagcc....................................................................  101     0   Mm2
.....................uuugauuguugcucagaaagUc....................................................................  1       1   Mm2
.....................Auugauuguugcucagaaagcc....................................................................  1       1   Mm2
.....................uuugUuuguugcucagaaagcc....................................................................  1       1   Mm2
.....................uuugauuguugcCcagaaagcc....................................................................  1       1   Mm2
.....................uuugauuguugcucUgaaagcc....................................................................  1       1   Mm2
.....................uuugauuguuUcucagaaagcc....................................................................  8       1   Mm2
.....................uuuAauuguugcucagaaagcc....................................................................  1       1   Mm2
.....................uuugauuguugcucagaaagccU...................................................................  140     1   Mm2
.....................uuugauuguugcucagaaagccg...................................................................  5       0   Mm2
.....................uuugauuguugcucagaaagccC...................................................................  2       1   Mm2
```

```
                    Mature                        Star
caaagagucguuuguccguguuuugauuguugcucagaaagccguccauuuaagggccuucgaguagcaaucaaauucagaggggaacgacgcuucaacauaccugauuac
.....................uuugauuguugcucagaaaAccg...................................................................	1	1	Mm2
......................uugauuguugcucagaaagcc....................................................................	9	0	Mm2
......................uugauuguugcucagaaagccU...................................................................	8	1	Mm2
.......................ugauuguugcucagaaagcc....................................................................	1	0	Mm2
.......................ugauuguugcucagaaagccU...................................................................	3	1	Mm2
........................................................ccuucgaguagcaaucaaauuc.................................	13	0	Mm2
........................................................ccuucgaguagcaaucUaauuc.................................	1	1	Mm2
........................................................ccuucgaguagcaaucaaauuca................................	40	0	Mm2
..........................................................uucgaguagcaaucaaauucagU..............................	1	1	Mm2

...................Cuuuugauuguugcucagaaagcc....................................................................	14	1	Mm1
...................Cuuuugauuguugcucagaaagccg...................................................................	1	1	Mm1
....................uuuugauuguugcucaga.........................................................................	8	0	Mm1
....................uuuugauuguAgcucagaaa.......................................................................	1	1	Mm1
....................uuuugauuguugcucagCaa.......................................................................	1	1	Mm1
....................uuuugauuguugcucagaaU.......................................................................	3	1	Mm1
....................uuuugauuguugcucagUaa.......................................................................	1	1	Mm1
....................uuuugauuguugcucagaaa.......................................................................	34	0	Mm1
....................uuuugauuguugcucagCaag......................................................................	4	1	Mm1
....................uuuugauuguugcucagUaag......................................................................	3	1	Mm1
....................uuGugauuguugcucagaaag......................................................................	2	1	Mm1
....................uuuugauuguCgcucagaaag......................................................................	1	1	Mm1
....................uuuugauuguugcucGgaaag......................................................................	1	1	Mm1
....................uuuuCauuguugcucagaaag......................................................................	1	1	Mm1
....................uuuugauuguugcucagaaaA......................................................................	4	1	Mm1
....................uuuugauuguugcucagaaaU......................................................................	8	1	Mm1
....................uuuugauuguugcucaCaaag......................................................................	1	1	Mm1
....................uuuugauuguugcucagaaag......................................................................	364	0	Mm1
....................uuuugauuguugcucagGaagc.....................................................................	11	1	Mm1
....................uuuugauuguugcucaUaaagc.....................................................................	3	1	Mm1
....................uuuugauuguugcucagUaagc.....................................................................	7	1	Mm1
....................uuuugauuguugcucagaaaAc.....................................................................	1	1	Mm1
....................uuuugauuguugcucaAaaagc.....................................................................	1	1	Mm1
....................uuuugauuguugcucGgaaagc.....................................................................	1	1	Mm1
....................uuuugauuguugcucaCaaagc.....................................................................	9	1	Mm1
....................uuuugauugAugcucagaaagc.....................................................................	1	1	Mm1
....................uuuugauuguugcucagaaagc.....................................................................	1740	0	Mm1
....................uuuCgauuguugcucagaaagc.....................................................................	1	1	Mm1
....................uuuugauuguugcuUagaaagc.....................................................................	2	1	Mm1
....................uuuugauuguCgcucagaaagc.....................................................................	2	1	Mm1
....................uuuugauuguugcucagaaagA.....................................................................	6	1	Mm1
....................uuuugauuguugcucagCaagc.....................................................................	7	1	Mm1
....................uuuugauuguugcucagaaaUc.....................................................................	1	1	Mm1
....................uAuugauuguugcucagaaagc.....................................................................	3	1	Mm1
....................uuuugauuguugcucagaaGgc.....................................................................	3	1	Mm1
....................uuuugauuguAgcucagaaagc.....................................................................	3	1	Mm1
....................uuuugauuguugAucagaaagc.....................................................................	1	1	Mm1
....................uuGugauuguugcucagaaagc.....................................................................	3	1	Mm1
....................Cuuugauuguugcucagaaagc.....................................................................	6	1	Mm1
....................uuuuAauuguugcucagaaagc.....................................................................	1	1	Mm1
....................uuCugauuguugcucagaaagc.....................................................................	1	1	Mm1
....................uuuugauuguugcucagaaagU.....................................................................	24	1	Mm1
....................uuuugauuguGgcucagaaagc.....................................................................	2	1	Mm1
....................uuuugauuguugcGcagaaagcc....................................................................	1	1	Mm1
....................uuuugauuguugcCcagaaagcc....................................................................	2	1	Mm1
....................uuuGgauuguugcucagaaagcc....................................................................	1	1	Mm1
....................uuuugauuguugcucagaaUgcc....................................................................	1	1	Mm1
....................uuuugauuAuugcucagaaagcc....................................................................	2	1	Mm1
....................uuuugauuguugcucaUaaagcc....................................................................	51	1	Mm1
....................Guuugauuguugcucagaaagcc....................................................................	1	1	Mm1
....................uuuugauuguugcucagCaagcc....................................................................	77	1	Mm1
....................uuuugauuguuUcucagaaagcc....................................................................	7	1	Mm1
....................uuuuAauuguugcucagaaagcc....................................................................	2	1	Mm1
....................uuuugauuguCgcucagaaagcc....................................................................	26	1	Mm1
....................uuAugauuguugcucagaaagcc....................................................................	5	1	Mm1
....................uuuuUauuguugcucagaaagcc....................................................................	2	1	Mm1
....................uuGugauuguugcucagaaagcc....................................................................	10	1	Mm1
....................uGuugauuguugcucagaaagcc....................................................................	4	1	Mm1
....................uuuugauuguugcucagaaagUc....................................................................	1	1	Mm1
....................Nuuugauuguugcucagaaagcc....................................................................	12	1	Mm1
```

```
                    Mature                          Star
caaagagucguuuguccguguuuugauuguugcucagaaagccguccauuuaagggccuucgaguagcaaucaaauucagaggggaacgacgcuucaacauaccugauuac
....................uuuugauuguugcucagaaagcU....................................................................    119     1    Mm1
....................uuuCgauuguugcucagaaagcc....................................................................    4       1    Mm1
....................uuuugauuguugUucagaaagcc....................................................................    5       1    Mm1
....................uuuugauugCugcucagaaagcc....................................................................    2       1    Mm1
....................uCuugauuguugcucagaaagcc....................................................................    5       1    Mm1
....................uAuugauuguugcucagaaagcc....................................................................    10      1    Mm1
....................uuuugauuguugAucagaaagcc....................................................................    2       1    Mm1
....................uuuugauuguugcuUagaaagcc....................................................................    14      1    Mm1
....................uuuugauuguugcucUgaaagcc....................................................................    3       1    Mm1
....................uuuugauuguugcucagUaagcc....................................................................    72      1    Mm1
....................uuuugauuguugcucagaaagcc....................................................................    21719   0    Mm1
....................uuuugauuguugcucagaaaCcc....................................................................    2       1    Mm1
....................uuuugUuuguugcucagaaagcc....................................................................    8       1    Mm1
....................uuuugNuuguugcucagaaagcc....................................................................    3       1    Mm1
....................uuuugauuguugcucagaaCgcc....................................................................    1       1    Mm1
....................Cuuugauuguugcucagaaagcc....................................................................    12      1    Mm1
....................uuuugauuguugcucaAaaagcc....................................................................    47      1    Mm1
....................uuuugauuguugcucagaaagAc....................................................................    3       1    Mm1
....................uuuugaAuguugcucagaaagcc....................................................................    1       1    Mm1
....................uuuugauuguugGucagaaagcc....................................................................    1       1    Mm1
....................uuuugauuguugcucagaaagGc....................................................................    1       1    Mm1
....................uuuugauuguugcucagaGagcc....................................................................    4       1    Mm1
....................uuCugauuguugcucagaaagcc....................................................................    1       1    Mm1
....................uuuugauuguugcucagaaaAcc....................................................................    6       1    Mm1
....................uuuugauuguugcucagaUagcc....................................................................    2       1    Mm1
....................uuuugauuguugcucagaaagcA....................................................................    30      1    Mm1
....................uuuugauuguGgcucagaaagcc....................................................................    32      1    Mm1
....................uuuugauAguugcucagaaagcc....................................................................    2       1    Mm1
....................uuuugauGguugcucagaaagcc....................................................................    2       1    Mm1
....................uuuugaCuguugcucagaaagcc....................................................................    5       1    Mm1
....................Auuugauuguugcucagaaagcc....................................................................    6       1    Mm1
....................uuuugauugGugcucagaaagcc....................................................................    1       1    Mm1
....................uuuugauuUuugcucagaaagcc....................................................................    7       1    Mm1
....................uuuugauuguugcucGgaaagcc....................................................................    1       1    Mm1
....................uuuugauuguugcucagaaaUcc....................................................................    5       1    Mm1
....................uuuCauuguugcucagaaagcc....................................................................    1       1    Mm1
....................uuuugauCguugcucagaaagcc....................................................................    1       1    Mm1
....................uuuugauuguAgcucagaaagcc....................................................................    53      1    Mm1
....................uuuugauugAugcucagaaagcc....................................................................    3       1    Mm1
....................uuuugaNuguugcucagaaagcc....................................................................    1       1    Mm1
....................uuuugauuguugcucagGaagcc....................................................................    71      1    Mm1
....................uuuugGuuguugcucagaaagcc....................................................................    2       1    Mm1
....................uuuugauuguugcAcagaaagcc....................................................................    4       1    Mm1
....................uuuugauuguugcucagaaGgcc....................................................................    17      1    Mm1
....................uuuugauuguugcucaCaaagcc....................................................................    69      1    Mm1
....................uuuugauuguugcucagaaaUccg...................................................................    1       1    Mm1
....................uuuugauuguugcucagaaagccC...................................................................    9       1    Mm1
....................uuuugauuguugcucagaaagccU...................................................................    81509   1    Mm1
....................uuuugauuguugcucagaaagccg...................................................................    180     0    Mm1
....................uuuugauuguugcucagaaagccA...................................................................    35      1    Mm1
....................uuuugauuguAgcucagaaagccg...................................................................    1       1    Mm1
....................uuuugauuguugcucaCaaagccg...................................................................    1       1    Mm1
....................uuuugauuguugcucagGaagccg...................................................................    1       1    Mm1
....................uuuugauuguugcucagaaaCccg...................................................................    1       1    Mm1
....................Cuuugauuguugcucagaaagccg...................................................................    1       1    Mm1
....................uuuugauuguugcucagUaagccg...................................................................    1       1    Mm1
....................uuuugauuguugcuAagaaagccg...................................................................    1       1    Mm1
....................uuuugaAuguugcucagaaagccg...................................................................    1       1    Mm1
....................uuuugauuguugcucaUaaagccg...................................................................    1       1    Mm1
....................uuuugauuguugcGcagaaagccg...................................................................    1       1    Mm1
....................uGuugauuguugcucagaaagccg...................................................................    1       1    Mm1
....................uuuugauuguugcucagaaagccCu..................................................................    3       1    Mm1
....................uuuugauuguugcucagaaagccUu..................................................................    25      1    Mm1
.....................uuugauuguuAcucagaaagcc....................................................................    1       1    Mm1
.....................uuugauuguugcucagaaagcc....................................................................    48      0    Mm1
.....................uuugauuguugcucagaaagccU...................................................................    69      1    Mm1
......................uugauuguugcucagaaagccU...................................................................    3       1    Mm1

..................uCuuuugauuguugcucagaaagcc....................................................................    18      1    Fm1
..................uCuuuugauuguugcucagaaagccg...................................................................    2       1    Fm1
```

Mature **Star**

caaagagucguuuguccgug uuuugauuguugcucagaaagccg uccauuuaaggg ccuucgaguagcaaucaaauuca gaggggaacgacgcuucaacauaccugauuac			
...................Cuuuugauuguugcucagaaag..	4	1	Fm1
...................Cuuuugauuguugcucagaaagc...	14	1	Fm1
...................Cuuuugauuguugcucagaaagcc..	383	1	Fm1
...................guuuugauuguugcucagaaagccU...	14	1	Fm1
...................Cuuuugauuguugcucagaaagccg...	44	1	Fm1
....................uuuugauuguugcucaga...	30	0	Fm1
....................uuuugauCguugcucagaa..	1	1	Fm1
....................uuuugauuguugcucagGa..	1	1	Fm1
....................uuuugauuguugcucagCa..	1	1	Fm1
....................uuuugauuguugcucaCaa..	1	1	Fm1
....................uuuugauuguugcucagaa..	72	0	Fm1
....................uuuugauuguugcucagaU..	5	1	Fm1
....................uuuugauuguAgcucagaaa...	1	1	Fm1
....................uuuugauAguugcucagaaa...	1	1	Fm1
....................uuuugauuguugcucaCaaa...	1	1	Fm1
....................uuuugauuguugcucagGaa...	1	1	Fm1
....................uuuugauuguugcucagaaa...	179	0	Fm1
....................uuuugauuguugcucagCaa...	1	1	Fm1
....................uuuugauuguugcucagaaU...	74	1	Fm1
....................uuuugauuguugcucagaaG...	13	1	Fm1
....................uuuugauuguugcucaAaaa...	1	1	Fm1
....................Nuuugauuguugcucagaaa...	1	1	Fm1
....................uuuugauuAuugcucagaaa...	1	1	Fm1
....................uuuugauuguugcucagCaag..	26	1	Fm1
....................Nuuugauuguugcucagaaag..	3	1	Fm1
....................uuuugauAguugcucagaaag..	3	1	Fm1
....................uuuugauuguugcucagaaUg..	2	1	Fm1
....................uuuugauuUuugcucagaaag..	2	1	Fm1
....................uuuugauuguugUucagaaag..	1	1	Fm1
....................uuuugauuguugcucagaaag..	6965	0	Fm1
....................uuuugauuguuUcucagaaag..	1	1	Fm1
....................uuuugauuguAgcucagaaag..	20	1	Fm1
....................uuuuCauuguugcucagaaag..	2	1	Fm1
....................Auuugauuguugcucagaaag..	1	1	Fm1
....................uuAugauuguugcucagaaag..	2	1	Fm1
....................uuuugauuguugcucaUaaag..	13	1	Fm1
....................uAuugauuguugcucagaaag..	4	1	Fm1
....................uuuugauuguugcucagaaGg..	5	1	Fm1
....................uuuugauuguugcucagaaaA..	8	1	Fm1
....................uuuugauuguCgcucagaaag..	9	1	Fm1
....................uuuugauuguugcucagGaag..	21	1	Fm1
....................uuuugauuguuAcucagaaag..	1	1	Fm1
....................uuuugauuguugcuAagaaag..	1	1	Fm1
....................uuuugauugGugcucagaaag..	1	1	Fm1
....................uuuugauuguugcAcagaaag..	1	1	Fm1
....................uuuugauugCugcucagaaag..	1	1	Fm1
....................uuuugaAuguugcucagaaag..	1	1	Fm1
....................uuuuUauuguugcucagaaag..	1	1	Fm1
....................uuuugGuuguugcucagaaag..	3	1	Fm1
....................uuGugauuguugcucagaaag..	5	1	Fm1
....................uuuugauuAuugcucagaaag..	1	1	Fm1
....................uuuugauuguugcucaCaaag..	13	1	Fm1
....................uuuCgauuguugcucagaaag..	2	1	Fm1
....................uuCugauuguugcucagaaag..	3	1	Fm1
....................uCuugauuguugcucagaaag..	3	1	Fm1
....................uuuugauuguugcucagaUag..	2	1	Fm1
....................Cuuugauuguugcucagaaag..	2	1	Fm1
....................uuuugauuguugcucagaaaU..	16	1	Fm1
....................uuuugauuguugcucagaaCg..	1	1	Fm1
....................uuuugauuguugcucagUaag..	27	1	Fm1
....................uuuugNuuguugcucagaaag..	2	1	Fm1
....................uGuugauuguugcucagaaag..	1	1	Fm1
....................uuuugauuguGgcucagaaag..	5	1	Fm1
....................uuuugauuguugcuGagaaag..	1	1	Fm1
....................uuuugauuguugcucaAaaag..	9	1	Fm1
....................uuuugUuuguugcucagaaag..	1	1	Fm1
....................uuuugauuguugcucagaaagA...	61	1	Fm1
....................uuuugauuguugcucagGaagc...	110	1	Fm1
....................uuuugaNuguugcucagaaagc...	4	1	Fm1
....................uuuuCauuguugcucagaaagc...	2	1	Fm1

```
                    Mature                          Star
caaagagucguuuguccguguuuugauuguugcucagaaagccguccauuuaagggccuucgaguagcaaucaaauucagaggggaacgacgcuucaacauaccugauuac
....................uuuugaCuguugcucagaaagc.....................................................................   6      1   Fm1
....................uuuuUauuguugcucagaaagc.....................................................................   4      1   Fm1
....................uuuAgauuguugcucagaaagc.....................................................................   5      1   Fm1
....................uuuuAauuguugcucagaaagc.....................................................................   2      1   Fm1
....................uAuugauuguugcucagaaagc.....................................................................   9      1   Fm1
....................uuuugauuguugcucaCaaagc.....................................................................   91     1   Fm1
....................uuuugauuguugcucagaaUgc.....................................................................   2      1   Fm1
....................Cuuugauuguugcucagaaagc.....................................................................   15     1   Fm1
....................uuuugauuguugcucagaaagc.....................................................................   34042  0   Fm1
....................uuuugauuguugcuGagaaagc.....................................................................   4      1   Fm1
....................uuuugauuguugcucagaaCgc.....................................................................   1      1   Fm1
....................uuuugauuguugcucagaaaAc.....................................................................   1      1   Fm1
....................uuuugauuguugcucaUaaagc.....................................................................   61     1   Fm1
....................uuuugauuguuAcucagaaagc.....................................................................   5      1   Fm1
....................uuuugauuguuUcucagaaagc.....................................................................   12     1   Fm1
....................uuuugauuguugcuUagaaagc.....................................................................   21     1   Fm1
....................Nuuugauuguugcucagaaagc.....................................................................   20     1   Fm1
....................uuuugauuguugcucaAaaagc.....................................................................   57     1   Fm1
....................uuuugauuguAgcucagaaagc.....................................................................   60     1   Fm1
....................uuuugauuguugcucagaGagc.....................................................................   6      1   Fm1
....................uuuugauuguugAucagaaagc.....................................................................   3      1   Fm1
....................uuuCgauuguugcucagaaagc.....................................................................   1      1   Fm1
....................uuuugauuguugcucagaUagc.....................................................................   4      1   Fm1
....................uuuugauuguugcucagCaagc.....................................................................   131    1   Fm1
....................uuuugauCguugcucagaaagc.....................................................................   5      1   Fm1
....................uuuugauugCugcucagaaagc.....................................................................   4      1   Fm1
....................Guuugauuguugcucagaaagc.....................................................................   3      1   Fm1
....................uuuugauugAugcucagaaagc.....................................................................   2      1   Fm1
....................uuuugNuuguugcucagaaagc.....................................................................   2      1   Fm1
....................uuuugauuguugcGcagaaagc.....................................................................   2      1   Fm1
....................uuuugauuAuugcucagaaagc.....................................................................   6      1   Fm1
....................uuuugauuUuugcucagaaagc.....................................................................   4      1   Fm1
....................uuuugauuguugcAcagaaagc.....................................................................   6      1   Fm1
....................uuuugaAuguugcucagaaagc.....................................................................   5      1   Fm1
....................uuuugauuguugcucagaaGgc.....................................................................   13     1   Fm1
....................uuuugauuguugcuAagaaagc.....................................................................   3      1   Fm1
....................uuuugauuguugcucCgaaagc.....................................................................   3      1   Fm1
....................uuuugauuguugcucGgaaagc.....................................................................   6      1   Fm1
....................uuuugauuguGgcucagaaagc.....................................................................   39     1   Fm1
....................uuuugauuguugcucagaCagc.....................................................................   1      1   Fm1
....................uCuugauuguugcucagaaagc.....................................................................   13     1   Fm1
....................Auuugauuguugcucagaaagc.....................................................................   1      1   Fm1
....................uuuugauuguugGucagaaagc.....................................................................   1      1   Fm1
....................uuuugauuguugcucagaaaUc.....................................................................   8      1   Fm1
....................uuuugauuguCgcucagaaagc.....................................................................   38     1   Fm1
....................uuuugUuuguugcucagaaagc.....................................................................   11     1   Fm1
....................uuuugauuguugcucagaaagU.....................................................................   188    1   Fm1
....................uuuugauuguugcCcagaaagc.....................................................................   5      1   Fm1
....................uGuugauuguugcucagaaagc.....................................................................   3      1   Fm1
....................uuuugGuuguugcucagaaagc.....................................................................   1      1   Fm1
....................uuuugauuguugcucagaaagG.....................................................................   3      1   Fm1
....................uuuugauuguugcucagUaagc.....................................................................   113    1   Fm1
....................uuuugauuguugUucagaaagc.....................................................................   17     1   Fm1
....................uuuugauuguugcucUgaaagc.....................................................................   4      1   Fm1
....................uuuugCuuguugcucagaaagc.....................................................................   3      1   Fm1
....................uuAugauuguugcucagaaagc.....................................................................   7      1   Fm1
....................uuuugauAguugcucagaaagc.....................................................................   2      1   Fm1
....................uuCugauuguugcucagaaagc.....................................................................   5      1   Fm1
....................uuuugauGguugcucagaaagc.....................................................................   1      1   Fm1
....................uuuugauugGugcucagaaagc.....................................................................   3      1   Fm1
....................uuuugauuCuugcucagaaagc.....................................................................   1      1   Fm1
....................uuGugauuguugcucagaaagc.....................................................................   14     1   Fm1
....................uuuugauAguugcucagaaagcc....................................................................   86     1   Fm1
....................uuuugauuguugcucagaaagcA....................................................................   687    1   Fm1
....................uuCugauuguugcucagaaagcc....................................................................   133    1   Fm1
....................uAuugauuguugcucagaaagcc....................................................................   304    1   Fm1
....................uuuugCuuguugcucagaaagcc....................................................................   41     1   Fm1
....................uuuugaCuguugcucagaaagcc....................................................................   88     1   Fm1
....................uuuugauCguugcucagaaagcc....................................................................   125    1   Fm1
....................uuuugauuguugcucagNaagcc....................................................................   1      1   Fm1
```

Mature Star

caaagagucguuuguccgug**uuuugauuguugcucagaaagccg**uccauuuaaggg**ccuucgaguagcaaucaaauuca**gaggggaacgacgcuucaacauaccugauuac			
....................uuuugGuuguugcucagaaagcc..	93	1	Fm1
....................uuuugauuguugcucaCaaagcc..	2414	1	Fm1
....................uuuugauuguugcucagaaagcU..	2290	1	Fm1
....................uuuugauuguugcucagaUagcc..	47	1	Fm1
....................uuuugauuguugcucagaaCgcc..	25	1	Fm1
....................uuuugauuguugcucagaaagGc..	29	1	Fm1
....................uuuGgauuguugcucagaaagcc..	30	1	Fm1
....................uuuuAauuguugcucagaaagcc..	56	1	Fm1
....................uuuugauuguugcucaAaaagcc..	1392	1	Fm1
....................uuuugauuguugcucagaaaCcc..	35	1	Fm1
....................uuuugauuguAgcucagaaagcc..	1539	1	Fm1
....................uuuAgauuguugcucagaaagcc..	111	1	Fm1
....................uuuugauuguCgcucagaaagcc..	857	1	Fm1
....................uuuugauuguuAcucagaaagcc..	124	1	Fm1
....................uuuugaGuguugcucagaaagcc..	43	1	Fm1
....................uuuugauuguugcucagaaaUcc..	171	1	Fm1
....................uuuugauuguugcucagaaUgcc..	42	1	Fm1
....................uuNugauuguugcucagaaagcc..	4	1	Fm1
....................uuuugauuguugcucagaaagcc..	789906	0	Fm1
....................uuuugauuguugGucagaaagcc..	32	1	Fm1
....................uuuCgauuguugcucagaaagcc..	158	1	Fm1
....................uNuugauuguugcucagaaagcc..	7	1	Fm1
....................uuuugUuuguugcucagaaagcc..	258	1	Fm1
....................uuuugauGguugcucagaaagcc..	38	1	Fm1
....................uuAugauuguugcucagaaagcc..	151	1	Fm1
....................uuuugNuuguugcucagaaagcc..	114	1	Fm1
....................Cuuugauuguugcucagaaagcc..	358	1	Fm1
....................Guuugauuguugcucagaaagcc..	20	1	Fm1
....................uuuugauuUuugcucagaaagcc..	138	1	Fm1
....................uuuugauuguugUucagaaagcc..	132	1	Fm1
....................uuuugauuguugcucaUaaagcc..	1717	1	Fm1
....................uuuugauuguugcCcagaaagcc..	125	1	Fm1
....................uuuugauuguugcucagaaaAcc..	128	1	Fm1
....................uuuugauuguugcucagaGagcc..	80	1	Fm1
....................uuGugauuguugcucagaaagcc..	271	1	Fm1
....................uuuugauuguugcucagaaagcG..	111	1	Fm1
....................uuuugauuAuugcucagaaagcc..	64	1	Fm1
....................uuuugauuguGgcucagaaagcc..	989	1	Fm1
....................uuuugauuguugcucGgaaagcc..	118	1	Fm1
....................uGuugauuguugcucagaaagcc..	86	1	Fm1
....................uuuugauugGugcucagaaagcc..	37	1	Fm1
....................uuuuCauuguugcucagaaagcc..	53	1	Fm1
....................uuuugauuguugcAcagaaagcc..	144	1	Fm1
....................uuuugauuguuUcucagaaagcc..	224	1	Fm1
....................uuuugauugAugcucagaaagcc..	70	1	Fm1
....................uuuugauuguugcucagCaagcc..	3162	1	Fm1
....................uuuugauuguugcuAagaaagcc..	52	1	Fm1
....................uuuugauuguugcucCgaaagcc..	20	1	Fm1
....................uuuugauuguuCcucagaaagcc..	32	1	Fm1
....................Nuuugauuguugcucagaaagcc..	383	1	Fm1
....................uuuugauuguugcucagaCagcc..	12	1	Fm1
....................uuuugauuCuugcucagaaagcc..	23	1	Fm1
....................uuuugauugCugcucagaaagcc..	92	1	Fm1
....................uuuugauuguugcucUgaaagcc..	90	1	Fm1
....................uuuugauuguugcuGagaaagcc..	44	1	Fm1
....................uCuugauuguugcucagaaagcc..	215	1	Fm1
....................uuuuUauuguugcucagaaagcc..	48	1	Fm1
....................uuuugauuguugcucagUaagcc..	2419	1	Fm1
....................uuuugauuguugcucagaaagAc..	47	1	Fm1
....................uuuugaNuguugcucagaaagcc..	35	1	Fm1
....................Auuugauuguugcucagaaagcc..	85	1	Fm1
....................uuuugauuguugcGcagaaagcc..	48	1	Fm1
....................uuuugauuguugcucagaaagUc..	129	1	Fm1
....................uuuugauuguugcucagGaagcc..	2239	1	Fm1
....................uuuugauuguugcuUagaaagcc..	203	1	Fm1
....................uuuugauuguugAucagaaagcc..	73	1	Fm1
....................uuuugauuguugcucagaaGgcc..	479	1	Fm1
....................uuuugaAuguugcucagaaagcc..	58	1	Fm1
....................uuuCgauuguugcucagaaagccg...	23	1	Fm1
....................uuuugauAguugcucagaaagccg...	12	1	Fm1

```
                    Mature                    Star
caaagagucguuuguccguguuuugauuguugcucagaaagccguccauuuaagggccuucgaguagcaaucaaauucagaggggaacgacgcuucaacauaccugauuac
....................uuuugauuguugcucagaaagAcg...................................................................  11       1   Fm1
....................uuuugauuguugcuGagaaagccg...................................................................  7        1   Fm1
....................uuuugauuguugcucagaaagccg...................................................................  103960   0   Fm1
....................uuuugauuguugcucagaaagUcg...................................................................  16       1   Fm1
....................uuGugauuguugcucagaaagccg...................................................................  44       1   Fm1
....................uuuugauuguuCcucagaaagccg...................................................................  4        1   Fm1
....................uuuugauuguugcucagaaagGcg...................................................................  3        1   Fm1
....................uuuugauuguugcucCgaaagccg...................................................................  2        1   Fm1
....................uuuugauuguugcucagaaaUccg...................................................................  22       1   Fm1
....................uuuugauuguGgcucagaaagccg...................................................................  133      1   Fm1
....................uuuugauuguugUucagaaagccg...................................................................  24       1   Fm1
....................uuuugauuguugcucagGaagccg...................................................................  287      1   Fm1
....................uuuugaNuguugcucagaaagccg...................................................................  3        1   Fm1
....................uGuugauuguugcucagaaagccg...................................................................  6        1   Fm1
....................uuuugauuguugcucagCaagccg...................................................................  460      1   Fm1
....................uuuugaCuguugcucagaaagccg...................................................................  16       1   Fm1
....................uuuugauuguugcAcagaaagccg...................................................................  20       1   Fm1
....................Cuuugauuguugcucagaaagccg...................................................................  35       1   Fm1
....................uuuugauuguugcucGgaaagccg...................................................................  11       1   Fm1
....................uuuugauugAugcucagaaagccg...................................................................  12       1   Fm1
....................uuuugauuguugcucagaaGgccg...................................................................  65       1   Fm1
....................uuuugauuguugcucagaaagccN...................................................................  1        1   Fm1
....................uuuugauuguugcucagaaagccA...................................................................  7217     1   Fm1
....................uuuugauuguugcucagaaCgccg...................................................................  2        1   Fm1
....................uuuugauuguugcucaAaaagccg...................................................................  184      1   Fm1
....................uuuuUauuguugcucagaaagccg...................................................................  8        1   Fm1
....................uuuugauuguugcucagaaUgccg...................................................................  6        1   Fm1
....................uuuugaAuguugcucagaaagccg...................................................................  8        1   Fm1
....................uuAugauuguugcucagaaagccg...................................................................  26       1   Fm1
....................Nuuugauuguugcucagaaagccg...................................................................  53       1   Fm1
....................uuuugauCguugcucagaaagccg...................................................................  17       1   Fm1
....................uuuugauuguugcucagUaagccg...................................................................  332      1   Fm1
....................uuuugauuguugcucaUaaagccg...................................................................  201      1   Fm1
....................Auuugauuguugcucagaaagccg...................................................................  10       1   Fm1
....................uuCugauuguugcucagaaagccg...................................................................  16       1   Fm1
....................uuuugauuguugcucagaaagccC...................................................................  1313     1   Fm1
....................uuuugauuguugAucagaaagccg...................................................................  11       1   Fm1
....................uuuugUuuguugcucagaaagccg...................................................................  24       1   Fm1
....................uuuugauGguugcucagaaagccg...................................................................  4        1   Fm1
....................Guuugauuguugcucagaaagccg...................................................................  4        1   Fm1
....................uuuugGuuguugcucagaaagccg...................................................................  11       1   Fm1
....................uuuGgauuguugcucagaaagccg...................................................................  3        1   Fm1
....................uuuugauuAuugcucagaaagccg...................................................................  8        1   Fm1
....................uuuugCuuguugcucagaaagccg...................................................................  3        1   Fm1
....................uuuugauuguCgcucagaaagccg...................................................................  98       1   Fm1
....................uuuugaGuguugcucagaaagccg...................................................................  5        1   Fm1
....................uuuugauuguugcucagaaaAccg...................................................................  18       1   Fm1
....................uuuuAauuguugcucagaaagccg...................................................................  12       1   Fm1
....................uuuugNuuguugcucagaaagccg...................................................................  11       1   Fm1
....................uuuugauuguugcucaCaaagccg...................................................................  295      1   Fm1
....................uuuugauuguugcucagaaagccU...................................................................  1428247  1   Fm1
....................uuuAgauuguugcucagaaagccg...................................................................  9        1   Fm1
....................uuuugauuguugcCcagaaagccg...................................................................  21       1   Fm1
....................uuuuCauuguugcucagaaagccg...................................................................  3        1   Fm1
....................uuuugauuguuAcucagaaagccg...................................................................  17       1   Fm1
....................uuuugauuguugcucagaaaCccg...................................................................  5        1   Fm1
....................uuuugauuguugcGcagaaagccg...................................................................  6        1   Fm1
....................uuuugauuguugcucagaCagccg...................................................................  1        1   Fm1
....................uuuugauuguuUcucagaaagccg...................................................................  28       1   Fm1
....................uuuugauuCuugcucagaaagccg...................................................................  5        1   Fm1
....................uuuugauuguugcuUagaaagccg...................................................................  22       1   Fm1
....................uuuugauugGugcucagaaagccg...................................................................  2        1   Fm1
....................uAuugauuguugcucagaaagccg...................................................................  39       1   Fm1
....................uuuugauuguugcucagaaagcAg...................................................................  9        1   Fm1
....................uuuugauuguugcucagaaagcGg...................................................................  4        1   Fm1
....................uuuugauuUuugcucagaaagccg...................................................................  20       1   Fm1
....................uuuugauuguugcuAagaaagccg...................................................................  11       1   Fm1
....................uuuugauuguugGucagaaagccg...................................................................  2        1   Fm1
....................uuuugauugCugcucagaaagccg...................................................................  19       1   Fm1
....................uuuugauuguugcucagaGagccg...................................................................  16       1   Fm1
```

Mature **Star**

```
caaagagucguuuguccguguuuugauuguugcucagaaagccguccauuuaagggccuucgaguagcaaucaaauucagaggggaacgacgcuucaacauaccugauuac
....................uuuugauuguugcucagaaagcUg...................................................................   35     1   Fm1
....................uuNugauuguugcucagaaagccg...................................................................   1      1   Fm1
....................uCuugauuguugcucagaaagccg...................................................................   32     1   Fm1
....................uuuugauuguugcucagaUagccg...................................................................   2      1   Fm1
....................uuuugauuguAgcucagaaagccg...................................................................   196    1   Fm1
....................uuuugauuguugcucUgaaagccg...................................................................   11     1   Fm1
....................uuuugauuguugcucagaaagccAu..................................................................   85     1   Fm1
....................uuuugauuguugcucagaaagccgu..................................................................   1      0   Fm1
....................uuuugauuguugcucagaaagccCu..................................................................   25     1   Fm1
....................uuuugauuguugcucagaaagccUu..................................................................   1897   1   Fm1
....................uuuugauuguugcucagaaagccgucc................................................................   2      0   Fm1
.....................uuugauuguugcucagaaag......................................................................   8      0   Fm1
.....................uuugauuguugcucagaaagc.....................................................................   9      0   Fm1
.....................uuugauuguugcucagaGagc.....................................................................   1      1   Fm1
.....................uuuUauuguugcucagaaagc.....................................................................   2      1   Fm1
.....................uuugauuguugcucagaUagcc....................................................................   1      1   Fm1
.....................uuugauuguugcucagaGagcc....................................................................   3      1   Fm1
.....................uuugauuguugcucagaaaAcc....................................................................   4      1   Fm1
.....................uuugauuguugcucagaaagcc....................................................................   514    0   Fm1
.....................uuugauuguuAcucagaaagcc....................................................................   1      1   Fm1
.....................uuugauuguugcCcagaaagcc....................................................................   1      1   Fm1
.....................uuugauuguugcucaAaaagcc....................................................................   1      1   Fm1
.....................uuugauuguugcucagGaagcc....................................................................   1      1   Fm1
.....................uAugauuguugcucagaaagcc....................................................................   1      1   Fm1
.....................uuugauugGugcucagaaagcc....................................................................   1      1   Fm1
.....................uuugauuguugcucagCaagcc....................................................................   3      1   Fm1
.....................uuugCuuguugcucagaaagcc....................................................................   2      1   Fm1
.....................uuugauuguugcucagaaaUcc....................................................................   2      1   Fm1
.....................uuugauuguuUcucagaaagcc....................................................................   20     1   Fm1
.....................uuCgauuguugcucagaaagcc....................................................................   1      1   Fm1
.....................uuugauuUuugcucagaaagcc....................................................................   1      1   Fm1
.....................uuuUauuguugcucagaaagcc....................................................................   10     1   Fm1
.....................uuugGuuguugcucagaaagcc....................................................................   1      1   Fm1
.....................uuugauuguugcucagaaagcU....................................................................   1      1   Fm1
.....................uuugauuguugcucagaaagcA....................................................................   1      1   Fm1
.....................uuugauuguugcucagaaaAccg...................................................................   1      1   Fm1
.....................uCugauuguugcucagaaagccg...................................................................   1      1   Fm1
.....................uuugauuguugcuUagaaagccg...................................................................   1      1   Fm1
.....................uuuUauuguugcucagaaagccg...................................................................   2      1   Fm1
.....................uuugaAuguugcucagaaagccg...................................................................   1      1   Fm1
.....................uuugauuguugcucagaaagccU...................................................................   814    1   Fm1
.....................uuugauuguugcucagaaagccC...................................................................   3      1   Fm1
.....................uuugauuguugcucagaaagccA...................................................................   4      1   Fm1
.....................uuugauuguuUcucagaaagccg...................................................................   4      1   Fm1
.....................uuugauuguugcucagaaagccg...................................................................   56     0   Fm1
.....................uuugauuguugcucagaGagccg...................................................................   1      1   Fm1
.....................uuugauuguugcucagaaagccCu..................................................................   1      1   Fm1
.....................uuugauuguugcucagaaagccUu..................................................................   2      1   Fm1
......................uugauuguugcucagaaagc.....................................................................   2      0   Fm1
......................uuUauuguugcucagaaagcc....................................................................   2      1   Fm1
......................uugauuguugcucagaaagcc....................................................................   66     0   Fm1
......................uugauuguuUcucagaaagcc....................................................................   1      1   Fm1
......................uugauuguugcucagaGagcc....................................................................   1      1   Fm1
......................uugauuguugcucagaaCgcc....................................................................   1      1   Fm1
......................uugauuguugcucagaaagccg...................................................................   8      0   Fm1
......................uugauuguugcucagaaagccU...................................................................   65     1   Fm1
.......................ugauuguugcucagaaagc.....................................................................   1      0   Fm1
.......................ugauuguugcucagaaagcc....................................................................   4      0   Fm1
.......................ugauuguugcucagaaagccU...................................................................   2      1   Fm1
........................................................ccuucgaguagcaaucaaauuc.................................   4      0   Fm1
........................................................ccuucgaguagcaaucaaauuca................................   5      0   Fm1

..................uAuuuugauuguugcucagaaagc.....................................................................   1      1   Fm2
..................uCuuuugauuguugcucagaaagc.....................................................................   18     1   Fm2
..................uUuuuugauuguugcucagaaagccg...................................................................   1      1   Fm2
..................uCuuuugauuguugcucagaaagccg...................................................................   5      1   Fm2
...................guuuugauuguugcucaga.........................................................................   5      0   Fm2
...................Uuuuugauuguugcucagaaagcc....................................................................   2      1   Fm2
...................Cuuuugauuguugcucagaaagcc....................................................................   634    1   Fm2
...................Cuuuugauuguugcucagaaagccg...................................................................   116    1   Fm2
```

Mature Star

caaagagucguuuguccguguuuugauuguugcucagaaagccguccauuuaagggccuucgaguagcaaucaaauucagaggggaacgacgcuucaacauaccugauuac			
....................uuuugauuguugcucaga...	84	0	Fm2
....................uuuugauuguAgcucaga...	2	1	Fm2
....................uuuugauuguugcucagG...	3	1	Fm2
....................uuuugauuguugcucagU...	5	1	Fm2
....................uuuugauuguugcucaAa...	1	1	Fm2
....................uuuugauuguugcucagCa..	1	1	Fm2
....................uuuugauuguugcucagaa..	32	0	Fm2
....................uuuugauuguugcucagaaa...	338	0	Fm2
....................uuuugauuguugcucaAaaa...	1	1	Fm2
....................uuuugauuguCgcucagaaa...	1	1	Fm2
....................uuuugauuguugcucagUaa...	1	1	Fm2
....................uuuugGuuguugcucagaaa...	1	1	Fm2
....................uuuugauuguAgcucagaaa...	1	1	Fm2
....................uuuugauuguugcucagaaU...	26	1	Fm2
....................uuuugauuguugcucaCaaa...	1	1	Fm2
....................uuuugauuguugcAcagaaag..	1	1	Fm2
....................uuuugauuguugcucCgaaag..	1	1	Fm2
....................uuuugaCuguugcucagaaag..	1	1	Fm2
....................Auuugauuguugcucagaaag..	2	1	Fm2
....................uuuugauuguAgcucagaaag..	8	1	Fm2
....................uuuugauuguugcucagaaGg..	7	1	Fm2
....................uuuugauuguugcucaAaaag..	9	1	Fm2
....................uuuugaGuguugcucagaaag..	2	1	Fm2
....................uCuugauuguugcucagaaag..	1	1	Fm2
....................uuuugauuguugcucUgaaag..	2	1	Fm2
....................uuuugauuguugcucagaaaA..	23	1	Fm2
....................uGuugauuguugcucagaaag..	1	1	Fm2
....................uuuugauuguugcucagCaag..	23	1	Fm2
....................uuuugauuguuUcucagaaag..	2	1	Fm2
....................uuuugauuguuAcucagaaag..	1	1	Fm2
....................uuuugGuuguugcucagaaag..	2	1	Fm2
....................uuuugauuguugcuAagaaag..	1	1	Fm2
....................uuuugauuguGgcucagaaag..	6	1	Fm2
....................uuuugauuguugcCcagaaag..	4	1	Fm2
....................uuuugauuguugUucagaaag..	1	1	Fm2
....................uuuugauuguugcuUagaaag..	2	1	Fm2
....................uuAugauuguugcucagaaag..	1	1	Fm2
....................uuGugauuguugcucagaaag..	1	1	Fm2
....................uuuugNuuguugcucagaaag..	1	1	Fm2
....................uuuugauuguugcucagaaaU..	35	1	Fm2
....................uuuugUuuguugcucagaaag..	3	1	Fm2
....................uuuugauuguugcucagUaag..	22	1	Fm2
....................uuuugauuguugcucaUaaag..	7	1	Fm2
....................uuuugauuguCgcucagaaag..	10	1	Fm2
....................uuuugauuguugcucagaaag..	5460	0	Fm2
....................uuuCgauuguugcucagaaag..	1	1	Fm2
....................Nuuugauuguugcucagaaag..	3	1	Fm2
....................uuuugauuguugcucaCaaag..	19	1	Fm2
....................uuuuCauuguugcucagaaag..	1	1	Fm2
....................uuuugauuguugcucGgaaag..	3	1	Fm2
....................uuuugauCguugcucagaaag..	1	1	Fm2
....................uuuugauuguugAucagaaag..	1	1	Fm2
....................uuuugauuguugcucagGaag..	8	1	Fm2
....................uuuugauugCugcucagaaagc...	1	1	Fm2
....................uuuuAauuguugcucagaaagc...	2	1	Fm2
....................Guuugauuguugcucagaaagc...	6	1	Fm2
....................uuuugauuguugcucCgaaagc...	2	1	Fm2
....................uuuugauuguugcucagaaaAc...	6	1	Fm2
....................uuuugGuuguugcucagaaagc...	10	1	Fm2
....................uGuugauuguugcucagaaagc...	6	1	Fm2
....................uuuCgauuguugcucagaaagc...	12	1	Fm2
....................uuuugauuguugcucagaaagA...	83	1	Fm2
....................uuuugauuguugcuAagaaagc...	7	1	Fm2
....................uuuugauuguugcucagaaaCc...	8	1	Fm2
....................uuuugauuAuugcucagaaagc...	8	1	Fm2
....................uuuugauuguuUcucagaaagc...	35	1	Fm2
....................uuuugauuguugcucagaaagG...	3	1	Fm2
....................uuuugauuguugcucagaaaUc...	16	1	Fm2
....................uuuugauuguugcGcagaaagc...	5	1	Fm2
....................uuuugauuguugcucagaaagc...	60784	0	Fm2

```
                    Mature                          Star
caaagagucguuuguccguguuuugauuguugcucagaaagccguccauuuaagggccuucgaguagcaaucaaauucagaggggaacgacgcuucaacauaccugauuac
....................uuuugauuguugcucagaaagU.....................................................................    400     1     Fm2
....................uuAugauuguugcucagaaagc.....................................................................    12      1     Fm2
....................uuuugauuUuugcucagaaagc.....................................................................    6       1     Fm2
....................uuuugaNuguugcucagaaagc.....................................................................    2       1     Fm2
....................uuuugauuguuAcucagaaagc.....................................................................    10      1     Fm2
....................uAuugauuguugcucagaaagc.....................................................................    24      1     Fm2
....................uuuugauugAugcucagaaagc.....................................................................    4       1     Fm2
....................uuuugauugGugcucagaaagc.....................................................................    2       1     Fm2
....................uuuugauuguugcucagNaagc.....................................................................    1       1     Fm2
....................uuuugUuuguugcucagaaagc.....................................................................    42      1     Fm2
....................uuuugauuguugcucaCaaagc.....................................................................    180     1     Fm2
....................uuuugauAguugcucagaaagc.....................................................................    4       1     Fm2
....................uuuugauuguugcucagaaUgc.....................................................................    8       1     Fm2
....................uuuugauuguugcucGgaaagc.....................................................................    11      1     Fm2
....................uuuugCuuguugcucagaaagc.....................................................................    5       1     Fm2
....................uuuugauuguugcucUgaaagc.....................................................................    10      1     Fm2
....................uuuuCauuguugcucagaaagc.....................................................................    3       1     Fm2
....................uuuugauuguugcAcagaaagc.....................................................................    13      1     Fm2
....................uuuugauuguuCcucagaaagc.....................................................................    8       1     Fm2
....................Cuuugauuguugcucagaaagc.....................................................................    70      1     Fm2
....................uCuugauuguugcucagaaagc.....................................................................    12      1     Fm2
....................uuuugauuCuugcucagaaagc.....................................................................    7       1     Fm2
....................uuuugauuguugcucagaaCgc.....................................................................    5       1     Fm2
....................uuuAgauuguugcucagaaagc.....................................................................    2       1     Fm2
....................uuuugauuguugcucagUaagc.....................................................................    199     1     Fm2
....................uuGugauuguugcucagaaagc.....................................................................    20      1     Fm2
....................uuuGgauuguugcucagaaagc.....................................................................    4       1     Fm2
....................uuuugauCguugcucagaaagc.....................................................................    8       1     Fm2
....................uuuugNuuguugcucagaaagc.....................................................................    10      1     Fm2
....................uuuugauuguugcucagGaagc.....................................................................    171     1     Fm2
....................uuuugauuguugcuGagaaagc.....................................................................    6       1     Fm2
....................Nuuugauuguugcucagaaagc.....................................................................    30      1     Fm2
....................uuuugauuguugcCcagaaagc.....................................................................    18      1     Fm2
....................uuuugaGuguugcucagaaagc.....................................................................    1       1     Fm2
....................uuuugauuguugcucagaUagc.....................................................................    7       1     Fm2
....................uuuugauuguugcucagaaGgc.....................................................................    16      1     Fm2
....................uuuugauuguugAucagaaagc.....................................................................    2       1     Fm2
....................uuuugauuguugcucagaGagc.....................................................................    4       1     Fm2
....................uuuugauuguugUucagaaagc.....................................................................    9       1     Fm2
....................uuuuUauuguugcucagaaagc.....................................................................    5       1     Fm2
....................Auuugauuguugcucagaaagc.....................................................................    15      1     Fm2
....................uuCugauuguugcucagaaagc.....................................................................    8       1     Fm2
....................uuuugauuguCgcucagaaagc.....................................................................    69      1     Fm2
....................uuNugauuguugcucagaaagc.....................................................................    2       1     Fm2
....................uuuugauuguugcucaUaaagc.....................................................................    128     1     Fm2
....................uuuugauuguugcuUagaaagc.....................................................................    21      1     Fm2
....................uuuugauGguugcucagaaagc.....................................................................    2       1     Fm2
....................uuuugaCuguugcucagaaagc.....................................................................    9       1     Fm2
....................uuuugauuguGgcucagaaagc.....................................................................    80      1     Fm2
....................uuuugaAuguugcucagaaagc.....................................................................    4       1     Fm2
....................uuuugauuguAgcucagaaagc.....................................................................    133     1     Fm2
....................uuuugauuguugcucaAaaagc.....................................................................    99      1     Fm2
....................uuuugauuguugcucagCaagc.....................................................................    249     1     Fm2
....................uuuugaAuguugcucagaaagcc....................................................................    54      1     Fm2
....................uuuugauuguugcucagCaagcc....................................................................    3101    1     Fm2
....................uuuugauuguugcucaNaaagcc....................................................................    2       1     Fm2
....................uuuugauuguugUucagaaagcc....................................................................    178     1     Fm2
....................uuuugauuguugcucaCaaagcc....................................................................    2493    1     Fm2
....................uGuugauuguugcucagaaagcc....................................................................    116     1     Fm2
....................uuuugauuguugcucagaaagGc....................................................................    15      1     Fm2
....................uuuugauuguugcucagaCagcc....................................................................    13      1     Fm2
....................uuuugauuguugcuUagaaagcc....................................................................    215     1     Fm2
....................Guuugauuguugcucagaaagcc....................................................................    89      1     Fm2
....................uuuugauuguugcucaUaaagcc....................................................................    1686    1     Fm2
....................uuuugauuguugcucagaaaUcc....................................................................    200     1     Fm2
....................uuuugauuguugcucagaaaCcc....................................................................    48      1     Fm2
....................uuuugauuguugcucagUaagcc....................................................................    2458    1     Fm2
....................uNuugauuguugcucagaaagcc....................................................................    9       1     Fm2
....................uuNugauuguugcucagaaagcc....................................................................    8       1     Fm2
....................uuuugauuguugcucagaaCgcc....................................................................    22      1     Fm2
```

```
                    Mature                              Star
caaagagucguuuguccguguuuugauuguugcucagaaagccguccauuuaagggccuucgaguagcaaucaaauucagaggggaacgacgcuucaacauaccugauuac
....................uuuugauugGugcucagaaagcc....................................................................   24       1   Fm2
....................uuuugauuguugcucagaaUgcc....................................................................   49       1   Fm2
....................uuuugauuguGgcucagaaagcc....................................................................   997      1   Fm2
....................uuuuCauuguugcucagaaagcc....................................................................   41       1   Fm2
....................uuGugauuguugcucagaaagcc....................................................................   201      1   Fm2
....................Nuuugauuguugcucagaaagcc....................................................................   413      1   Fm2
....................uuuugaNuguugcucagaaagcc....................................................................   35       1   Fm2
....................uuuugaCuguugcucagaaagcc....................................................................   86       1   Fm2
....................uuuugauuguugcucGgaaagcc....................................................................   128      1   Fm2
....................uuuugauuguugcucagaaaAcc....................................................................   115      1   Fm2
....................uuuugauuguugcucaAaaagcc....................................................................   1400     1   Fm2
....................uAuugauuguugcucagaaagcc....................................................................   329      1   Fm2
....................uuuugNuuguugcucagaaagcc....................................................................   115      1   Fm2
....................uuAugauuguugcucagaaagcc....................................................................   115      1   Fm2
....................uuuugauuguugcucagaaagcU....................................................................   2290     1   Fm2
....................uuuugauuguugcucagaaagAc....................................................................   66       1   Fm2
....................uuuCgauuguugcucagaaagcc....................................................................   111      1   Fm2
....................uuuugauuguugcCcagaaagcc....................................................................   138      1   Fm2
....................uuuugauuguugcucCgaaagcc....................................................................   19       1   Fm2
....................uuuugauuguuUcucagaaagcc....................................................................   467      1   Fm2
....................Auuugauuguugcucagaaagcc....................................................................   168      1   Fm2
....................uuuugauuguuCcucagaaagcc....................................................................   55       1   Fm2
....................uuuugauuguugcucagaaagcG....................................................................   133      1   Fm2
....................uuuGgauuguugcucagaaagcc....................................................................   27       1   Fm2
....................uuuugauuguugcAcagaaagcc....................................................................   161      1   Fm2
....................uuuugauuguugcucagaaagcc....................................................................   827117   0   Fm2
....................uuuugauuguugcucagaaagcA....................................................................   1063     1   Fm2
....................uuuugauuUuugcucagaaagcc....................................................................   150      1   Fm2
....................uuuugauuguugcucagaaGgcc....................................................................   424      1   Fm2
....................uuuugauCguugcucagaaagcc....................................................................   118      1   Fm2
....................uuuuUauuguugcucagaaagcc....................................................................   51       1   Fm2
....................uuuuAauuguugcucagaaagcc....................................................................   67       1   Fm2
....................uuuugauuguugGucagaaagcc....................................................................   32       1   Fm2
....................uuuugauuguCgcucagaaagcc....................................................................   906      1   Fm2
....................uuuAgauuguugcucagaaagcc....................................................................   86       1   Fm2
....................uuuugauuguugcucagaGagcc....................................................................   108      1   Fm2
....................uCuugauuguugcucagaaagcc....................................................................   170      1   Fm2
....................uuuugauuguugcucagaaagUc....................................................................   96       1   Fm2
....................uuuugauuguugcGcagaaagcc....................................................................   54       1   Fm2
....................uuuugauuguugcuAagaaagcc....................................................................   91       1   Fm2
....................uuuugauAguugcucagaaagcc....................................................................   64       1   Fm2
....................uuuugauuguugcucagGaagcc....................................................................   2267     1   Fm2
....................uuuugauuguAgcucagaaagcc....................................................................   1691     1   Fm2
....................uuuugGuuguugcucagaaagcc....................................................................   143      1   Fm2
....................uuuugauugAugcucagaaagcc....................................................................   62       1   Fm2
....................uuuugauuguugAucagaaagcc....................................................................   73       1   Fm2
....................uuuugauuCuugcucagaaagcc....................................................................   36       1   Fm2
....................uuuugauugCugcucagaaagcc....................................................................   93       1   Fm2
....................uuuugCuuguugcucagaaagcc....................................................................   97       1   Fm2
....................uuuugauuguugcucUgaaagcc....................................................................   79       1   Fm2
....................uuuugUuuguugcucagaaagcc....................................................................   510      1   Fm2
....................uuCugauuguugcucagaaagcc....................................................................   124      1   Fm2
....................uuuugauuguugcuGagaaagcc....................................................................   42       1   Fm2
....................uuuugauuAuugcucagaaagcc....................................................................   64       1   Fm2
....................uuuugaGuguugcucagaaagcc....................................................................   39       1   Fm2
....................uuuugauuguuAcucagaaagcc....................................................................   157      1   Fm2
....................Cuuugauuguugcucagaaagcc....................................................................   636      1   Fm2
....................uuuugauuguugcucagaUagcc....................................................................   41       1   Fm2
....................uuuugauGguugcucagaaagcc....................................................................   34       1   Fm2
....................Guuugauuguugcucagaaagccg...................................................................   19       1   Fm2
....................uuuugauuguugcucagaaaCccg...................................................................   13       1   Fm2
....................uuGugauuguugcucagaaagccg...................................................................   39       1   Fm2
....................uuuugauuguugcucagaaagccA...................................................................   5752     1   Fm2
....................uuuugCuuguugcucagaaagccg...................................................................   24       1   Fm2
....................uuuugUuuguugcucagaaagccg...................................................................   133      1   Fm2
....................uuuugauuguugcucagaaagcUg...................................................................   67       1   Fm2
....................uuuugauuguugcucUgaaagccg...................................................................   18       1   Fm2
....................uuuugauugGugcucagaaagccg...................................................................   7        1   Fm2
....................uuuugauuguCgcucagaaagccg...................................................................   229      1   Fm2
....................uuuugauuguugcucagaCagccg...................................................................   4        1   Fm2
```

Mature Star

```
caaagagucguuuguccguguuuugauuguugcucagaaagccguccauuuaagggccuucgaguagcaaucaaauucagaggggaacgacgcuucaacauaccugauuac
....................uuuGgauuguugcucagaaagccg...................................................................   6        1   Fm2
....................uuuugauuguugcucaCaaagccg...................................................................   640      1   Fm2
....................uuuugauuguugcuUagaaagccg...................................................................   46       1   Fm2
....................uuuugauuguuGGucagaaagccg...................................................................   11       1   Fm2
....................uuuugauuguugcucagaaagcGg...................................................................   7        1   Fm2
....................uuuugauuguugcGcagaaagccg...................................................................   11       1   Fm2
....................Cuuugauuguugcucagaaagccg...................................................................   131      1   Fm2
....................uuuugauuguugcAcagaaagccg...................................................................   42       1   Fm2
....................uuuCgauuguugcucagaaagccg...................................................................   38       1   Fm2
....................uuuugauuguugcCcagaaagccg...................................................................   50       1   Fm2
....................uuuugauuguugcucagaaUgccg...................................................................   9        1   Fm2
....................uuuugauuguugcucagaaCgccg...................................................................   9        1   Fm2
....................uuuAgauuguugcucagaaagccg...................................................................   27       1   Fm2
....................uuuugauCguugcucagaaagccg...................................................................   25       1   Fm2
....................uuCugauuguugcucagaaagccg...................................................................   40       1   Fm2
....................uuuugauuguugcucagaaaUccg...................................................................   70       1   Fm2
....................uuuugauugCugcucagaaagccg...................................................................   22       1   Fm2
....................uCuugauuguugcucagaaagccg...................................................................   68       1   Fm2
....................uuuugauuguugcucagUaagccg...................................................................   637      1   Fm2
....................uuuugauuguugcucagaaagGcg...................................................................   5        1   Fm2
....................Nuuugauuguugcucagaaagccg...................................................................   113      1   Fm2
....................uuAugauuguugcucagaaagccg...................................................................   30       1   Fm2
....................uuuugaNuguugcucagaaagccg...................................................................   12       1   Fm2
....................uuuugauGguugcucagaaagccg...................................................................   10       1   Fm2
....................uuuuAauuguugcucagaaagccg...................................................................   23       1   Fm2
....................uuuuUauuguugcucagaaagccg...................................................................   15       1   Fm2
....................uuuugauuguugcucagaaagccU...................................................................   2941253  1   Fm2
....................uuuugauuguugcucagCaagccg...................................................................   877      1   Fm2
....................uuuugauugAugcucagaaagccg...................................................................   24       1   Fm2
....................uuuugaGuguugcucagaaagccg...................................................................   9        1   Fm2
....................uuuugauuguugcucagaaagccg...................................................................   212272   0   Fm2
....................uuuugauuguugcucagaaagccN...................................................................   1        1   Fm2
....................uuuugauuguugcucaUaaagccg...................................................................   428      1   Fm2
....................uuuugauuguugcuAagaaagccg...................................................................   26       1   Fm2
....................uuuugauAguugcucagaaagccg...................................................................   20       1   Fm2
....................uuuugauuguugcuGagaaagccg...................................................................   15       1   Fm2
....................uuuugauuguGgcucagaaagccg...................................................................   246      1   Fm2
....................uuuuCauuguugcucagaaagccg...................................................................   15       1   Fm2
....................uuuugNuuguugcucagaaagccg...................................................................   31       1   Fm2
....................uuuugauuguugcucagaGagccg...................................................................   32       1   Fm2
....................uuuugauuguugcucagaaGgccg...................................................................   94       1   Fm2
....................uuuugauuguugcucagaaagAcg...................................................................   19       1   Fm2
....................uNugauuguugcucagaaagccg...................................................................   2        1   Fm2
....................uGuugauuguugcucagaaagccg...................................................................   28       1   Fm2
....................uuuugauuCuugcucagaaagccg...................................................................   3        1   Fm2
....................uuuugauuguugcucagGaagccg...................................................................   638      1   Fm2
....................uuuugauuAuugcucagaaagccg...................................................................   27       1   Fm2
....................uuuugauuguuAcucagaaagccg...................................................................   36       1   Fm2
....................uuuugauuguugcucagaaagUcg...................................................................   19       1   Fm2
....................uuuugaAuguugcucagaaagccg...................................................................   22       1   Fm2
....................uuuugaCuguugcucagaaagccg...................................................................   18       1   Fm2
....................uAuugauuguugcucagaaagccg...................................................................   87       1   Fm2
....................uuuugauuguugcucagaaaAccg...................................................................   22       1   Fm2
....................uuuugauuguAgcucagaaagccg...................................................................   395      1   Fm2
....................uuuugauuguuUcucagaaagccg...................................................................   109      1   Fm2
....................Auuugauuguugcucagaaagccg...................................................................   36       1   Fm2
....................uuuugauuguugcucagaUagccg...................................................................   8        1   Fm2
....................uuuugauuguuCcucagaaagccg...................................................................   8        1   Fm2
....................uuuugauuguugcucGgaaagccg...................................................................   33       1   Fm2
....................uuuugauuguugcucaAaaagccg...................................................................   346      1   Fm2
....................uuuugGuuguugcucagaaagccg...................................................................   33       1   Fm2
....................uuuugauuguugcucCgaaagccg...................................................................   6        1   Fm2
....................uuuugauuguugcucagaaagcAg...................................................................   31       1   Fm2
....................uuuugauuUuugcucagaaagccg...................................................................   43       1   Fm2
....................uuuugauuguugAucagaaagccg...................................................................   15       1   Fm2
....................uuuugauuguugcucagaaagccC...................................................................   1093     1   Fm2
....................uuuugauuguugUucagaaagccg...................................................................   65       1   Fm2
....................uuuugauuguugcucagaaagccAu..................................................................   68       1   Fm2
....................uuuugauuguugcucagaaagccgA..................................................................   2        1   Fm2
....................uuuugauuguugcucagaaagccCu..................................................................   28       1   Fm2
```

```
                    Mature                              Star
caaagagucguuuguccguguuuugauuguugcucagaaagccguccauuuaagggccuucgaguagcaaucaaauucagaggggaacgacgcuucaacauaccugauuac
....................uuuugauuguugcucagaaagccUu..................................................................   2013   1   Fm2
....................uuuugauuguugcucagaaagccgucc................................................................   10     0   Fm2
.....................uuugauuguuUcucagaaagc.....................................................................   2      1   Fm2
.....................uuugauuguugcucagaaagc.....................................................................   18     0   Fm2
.....................uuuUauuguugcucagaaagc.....................................................................   1      1   Fm2
.....................uuAgauuguugcucagaaagcc....................................................................   1      1   Fm2
.....................uuuCauuguugcucagaaagcc....................................................................   2      1   Fm2
.....................uuugauuguuAcucagaaagcc....................................................................   2      1   Fm2
.....................uuugauuguugcucagUaagcc....................................................................   2      1   Fm2
.....................Nuugauuguugcucagaaagcc....................................................................   2      1   Fm2
.....................uuugauuguugGucagaaagcc....................................................................   1      1   Fm2
.....................uuuAauuguugcucagaaagcc....................................................................   1      1   Fm2
.....................uuugauuguugcucagaGagcc....................................................................   3      1   Fm2
.....................uuugGuuguugcucagaaagcc....................................................................   1      1   Fm2
.....................uuCgauuguugcucagaaagcc....................................................................   2      1   Fm2
.....................uuugaGuguugcucagaaagcc....................................................................   1      1   Fm2
.....................uuugauuguugcucagCaagcc....................................................................   1      1   Fm2
.....................uuuUauuguugcucagaaagcc....................................................................   11     1   Fm2
.....................Cuugauuguugcucagaaagcc....................................................................   1      1   Fm2
.....................uuugauuguugcucagaaagUc....................................................................   1      1   Fm2
.....................uuugauuUuugcucagaaagcc....................................................................   2      1   Fm2
.....................uuugauuguugcucagaaagcc....................................................................   423    0   Fm2
.....................uuugauuguuUcucagaaagcc....................................................................   30     1   Fm2
.....................uuugauuguugcucagaaaAcc....................................................................   3      1   Fm2
.....................uuugauuguuCcucagaaagcc....................................................................   1      1   Fm2
.....................uuAgauuguugcucagaaagccg...................................................................   1      1   Fm2
.....................uuugauuguugcucagUaagccg...................................................................   2      1   Fm2
.....................uuuUauuguugcucagaaagccg...................................................................   1      1   Fm2
.....................uuugaGuguugcucagaaagccg...................................................................   1      1   Fm2
.....................uuugauuguuUcucagaaagccg...................................................................   8      1   Fm2
.....................uuugauuguugcucagaaagccA...................................................................   5      1   Fm2
.....................uuugauuguugcucagaaagccU...................................................................   1169   1   Fm2
.....................uuugauuguugcucagaaagccC...................................................................   6      1   Fm2
.....................uuugauuguugcucagaaagccg...................................................................   112    0   Fm2
.....................uuuCauuguugcucagaaagccg...................................................................   1      1   Fm2
.....................uuugauuguugcucagaaagccUu..................................................................   1      1   Fm2
......................uuUauuguugcucagaaagc.....................................................................   1      1   Fm2
......................uugauuguugcCcagaaagcc....................................................................   1      1   Fm2
......................uuUauuguugcucagaaagcc....................................................................   1      1   Fm2
......................uugauuguuUcucagaaagcc....................................................................   1      1   Fm2
......................uugauuguugcucagaaagcc....................................................................   23     0   Fm2
......................uugauuguugcucagaaagccU...................................................................   91     1   Fm2
......................uugauuguuUcucagaaagccg...................................................................   2      1   Fm2
......................uugauuguugcucagaaagccg...................................................................   5      0   Fm2
.......................ugauuguugcucagaaagccU...................................................................   5      1   Fm2
........................................................ccuucgaguagcaaucaaauuca................................   22     0   Fm2
.........................................................cuucgaguagcaaucaaauucag...............................   5      0   Fm2

..................uCuuuugauuguugcucagaaagcc....................................................................   4      1   Mm3
...................guuuugauuguugcucagaaaU......................................................................   1      1   Mm3
...................guuuugauuguugcucagaaagcc....................................................................   1      0   Mm3
...................guuuugauuguugcucagaaagcU....................................................................   1      1   Mm3
...................Cuuuugauuguugcucagaaagcc....................................................................   12     1   Mm3
....................uuAugauuguugcucaga.........................................................................   1      1   Mm3
....................uuuugauuguugcucagU.........................................................................   3      1   Mm3
....................uuuugauuguugcucaga.........................................................................   15     0   Mm3
....................uuuugauuguugcucagaa........................................................................   23     0   Mm3
....................uuuugauuguugcucagaU........................................................................   1      1   Mm3
....................uuuugauuguugcucagCaa.......................................................................   1      1   Mm3
....................uuuugauuguugcucagaaa.......................................................................   119    0   Mm3
....................uuuugauuguugcucagaUa.......................................................................   1      1   Mm3
....................uuuugauuguugcucagaaU.......................................................................   21     1   Mm3
....................uuuugauuguugcucagUaa.......................................................................   1      1   Mm3
....................uuuugauuguugcucagaaGg......................................................................   1      1   Mm3
....................uuuugauuguugcucaUaaag......................................................................   1      1   Mm3
....................uuuugauuguugcucagaaag......................................................................   627    0   Mm3
....................uuuugauuguugcucaCaaag......................................................................   2      1   Mm3
....................uuuCgauuguugcucagaaag......................................................................   1      1   Mm3
....................uuuugauuguGgcucagaaag......................................................................   3      1   Mm3
....................uuuugauuguCgcucagaaag......................................................................   1      1   Mm3
```

Mature **Star**

```
caaagagucguuuguccguguuuugauuguugcucagaaagccguccauuuaagggccuucgaguagcaaucaaauucagaggggaacgacgcuucaacauaccugauuac
....................uuuugauuguugcucagaaaU......................................................................    12    1    Mm3
....................uuuugauuguugcucagGaag......................................................................    1     1    Mm3
....................uuuugauuguugcucagUaag......................................................................    3     1    Mm3
....................uuuugauuguugcucagCaag......................................................................    3     1    Mm3
....................uuuugauuguugcucagaaaA......................................................................    6     1    Mm3
....................uuuugauCguugcucagaaag......................................................................    1     1    Mm3
....................uuuugauuguugcucagaaGgc.....................................................................    2     1    Mm3
....................uuuugauuguugcucagaaagU.....................................................................    43    1    Mm3
....................uuuugauuguugcucagaaagc.....................................................................    4202  0    Mm3
....................uGuugauuguugcucagaaagc.....................................................................    1     1    Mm3
....................uuuugauuguugcucaAaaagc.....................................................................    12    1    Mm3
....................Guuugauuguugcucagaaagc.....................................................................    1     1    Mm3
....................uuuugauuguAgcucagaaagc.....................................................................    4     1    Mm3
....................uuuugauuguGgcucagaaagc.....................................................................    5     1    Mm3
....................uuGugauuguugcucagaaagc.....................................................................    2     1    Mm3
....................uuuugauuguugcucaUaaagc.....................................................................    9     1    Mm3
....................uuuugauuguugcucagaaCgc.....................................................................    1     1    Mm3
....................Nuuugauuguugcucagaaagc.....................................................................    2     1    Mm3
....................uuuugauuguuUcucagaaagc.....................................................................    2     1    Mm3
....................uuuugauuguugcucagaaUgc.....................................................................    1     1    Mm3
....................uuuugauuguCgcucagaaagc.....................................................................    7     1    Mm3
....................uuuugauuguugcucagGaagc.....................................................................    17    1    Mm3
....................Auuugauuguugcucagaaagc.....................................................................    2     1    Mm3
....................uuuugauCguugcucagaaagc.....................................................................    1     1    Mm3
....................uuuCgauuguugcucagaaagc.....................................................................    1     1    Mm3
....................uuuugauuUuugcucagaaagc.....................................................................    1     1    Mm3
....................uuuugauuguugcucagUaagc.....................................................................    13    1    Mm3
....................uuuugUuuguugcucagaaagc.....................................................................    2     1    Mm3
....................uuuugNuuguugcucagaaagc.....................................................................    1     1    Mm3
....................uuuugauuguugcucagaaaUc.....................................................................    1     1    Mm3
....................uuuugauuguugcucagaaagG.....................................................................    1     1    Mm3
....................uuuugauuguugcucUgaaagc.....................................................................    2     1    Mm3
....................Cuuugauuguugcucagaaagc.....................................................................    2     1    Mm3
....................uuuugauuguugcucagCaagc.....................................................................    15    1    Mm3
....................uuuugauuguugcucagaaagA.....................................................................    11    1    Mm3
....................uuuugauuguugcucagaaaAc.....................................................................    1     1    Mm3
....................uuuugauuguugcCcagaaagc.....................................................................    3     1    Mm3
....................uuuugauuguugcucaCaaagc.....................................................................    8     1    Mm3
....................uuuugauuguugcucagaGagc.....................................................................    1     1    Mm3
....................Nuuugauuguugcucagaaagcc....................................................................    21    1    Mm3
....................uuuugaGuguugcucagaaagcc....................................................................    4     1    Mm3
....................uuuugauuguugcAcagaaagcc....................................................................    12    1    Mm3
....................uuuGgauuguugcucagaaagcc....................................................................    1     1    Mm3
....................uuuugUuuguugcucagaaagcc....................................................................    18    1    Mm3
....................uuuugauugAugcucagaaagcc....................................................................    5     1    Mm3
....................uuuugauuguugcucagaaagcG....................................................................    7     1    Mm3
....................uuuugauuCuugcucagaaagcc....................................................................    1     1    Mm3
....................uuuugCuuguugcucagaaagcc....................................................................    2     1    Mm3
....................uuuugauugCugcucagaaagcc....................................................................    10    1    Mm3
....................uuuugauuUuugcucagaaagcc....................................................................    7     1    Mm3
....................uuuugauuguuCcucagaaagcc....................................................................    1     1    Mm3
....................uuuugauuguugAucagaaagcc....................................................................    5     1    Mm3
....................uuuugauuguugcucUgaaagcc....................................................................    4     1    Mm3
....................uuuugauuguugcucagaaagcA....................................................................    97    1    Mm3
....................uuuugauuguugcucagaaaUcc....................................................................    11    1    Mm3
....................uuuugauuguugcucGgaaagcc....................................................................    5     1    Mm3
....................uuuugauuguugcCcagaaagcc....................................................................    8     1    Mm3
....................uuuugauuguugGucagaaagcc....................................................................    2     1    Mm3
....................uuuugaNuguugcucagaaagcc....................................................................    1     1    Mm3
....................uuuugauuguugcucagUaagcc....................................................................    170   1    Mm3
....................uuuugaCuguugcucagaaagcc....................................................................    5     1    Mm3
....................uuuugauuguuUcucagaaagcc....................................................................    16    1    Mm3
....................uuuugaAuguugcucagaaagcc....................................................................    3     1    Mm3
....................uuuugauuguugcucagCaagcc....................................................................    212   1    Mm3
....................uuuugauuguugcucagaaCgcc....................................................................    3     1    Mm3
....................uuuugauuguAgcucagaaagcc....................................................................    103   1    Mm3
....................uuuugauuAuugcucagaaagcc....................................................................    1     1    Mm3
....................uuuugauuguGgcucagaaagcc....................................................................    70    1    Mm3
....................Guuugauuguugcucagaaagcc....................................................................    1     1    Mm3
....................uuCugauuguugcucagaaagcc....................................................................    9     1    Mm3
```

```
                    Mature                              Star
caaagagucguuuguccgugguuuugauuguugcucagaaagccguccauuuaagggccuucgaguagcaaucaaauucagaggggaacgacgcuucaacauaccugauuac
....................uuuugGuuguugcucagaaagcc....................................................................  5      1  Mm3
....................uuuugauuguugcucagaaagAc....................................................................  3      1  Mm3
....................uuuAgauuguugcucagaaagcc....................................................................  6      1  Mm3
....................uuuugauuguuAcucagaaagcc....................................................................  4      1  Mm3
....................Cuuugauuguugcucagaaagcc....................................................................  26     1  Mm3
....................uuuugauuguugcucagaaagcc....................................................................  56418  0  Mm3
....................uuuuCauuguugcucagaaagcc....................................................................  4      1  Mm3
....................uuuugauuguugUucagaaagcc....................................................................  11     1  Mm3
....................uGuugauuguugcucagaaagcc....................................................................  8      1  Mm3
....................uuuugauuguCgcucagaaagcc....................................................................  76     1  Mm3
....................uuuugauuguugcucagaaagcU....................................................................  305    1  Mm3
....................uuuugauugGugcucagaaagcc....................................................................  5      1  Mm3
....................uuuugauuguugcGcagaaagcc....................................................................  6      1  Mm3
....................uuuugauuguugcucagaUagcc....................................................................  3      1  Mm3
....................uuuugauuguugcucaUaaagcc....................................................................  116    1  Mm3
....................uuuugauuguugcucagaaaCcc....................................................................  3      1  Mm3
....................uuuugauuguugcucagaaGgcc....................................................................  72     1  Mm3
....................uuuugauGguugcucagaaagcc....................................................................  3      1  Mm3
....................uuuugauuguugcucaAaaagcc....................................................................  127    1  Mm3
....................uuuugauuguugcucCgaaagcc....................................................................  4      1  Mm3
....................uAuugauuguugcucagaaagcc....................................................................  27     1  Mm3
....................uuuugauuguugcuUagaaagcc....................................................................  13     1  Mm3
....................uuuugauuguugcucagaaagUc....................................................................  9      1  Mm3
....................uuuuUauuguugcucagaaagcc....................................................................  7      1  Mm3
....................Auuugauuguugcucagaaagcc....................................................................  4      1  Mm3
....................uuuCgauuguugcucagaaagcc....................................................................  10     1  Mm3
....................uuuugauuguugcucagaaaAcc....................................................................  6      1  Mm3
....................uuuugauuguugcuAagaaagcc....................................................................  2      1  Mm3
....................uuGugauuguugcucagaaagcc....................................................................  22     1  Mm3
....................uuuuAauuguugcucagaaagcc....................................................................  2      1  Mm3
....................uuuugauuguugcucagaGagcc....................................................................  3      1  Mm3
....................uuAugauuguugcucagaaagcc....................................................................  14     1  Mm3
....................uuuugauuguugcucagaaUgcc....................................................................  1      1  Mm3
....................uuuugauuguugcucaCaaagcc....................................................................  160    1  Mm3
....................uuuugauAguugcucagaaagcc....................................................................  7      1  Mm3
....................uuuugauCguugcucagaaagcc....................................................................  8      1  Mm3
....................uCuugauuguugcucagaaagcc....................................................................  11     1  Mm3
....................uuuugNuuguugcucagaaagcc....................................................................  8      1  Mm3
....................uuuugauuguugcucagGaagcc....................................................................  168    1  Mm3
....................Cuuugauuguugcucagaaagccg...................................................................  4      1  Mm3
....................uuuugauuguugcucagaaagccU...................................................................  98364  1  Mm3
....................uuuGgauuguugcucagaaagccg...................................................................  1      1  Mm3
....................uuuugauuguugcucagUaagccg...................................................................  6      1  Mm3
....................uuuCgauuguugcucagaaagccg...................................................................  1      1  Mm3
....................uuGugauuguugcucagaaagccg...................................................................  2      1  Mm3
....................uuuugauuguugcucagaaaUccg...................................................................  1      1  Mm3
....................uuuugauugCugcucagaaagccg...................................................................  1      1  Mm3
....................uuuugauuguuUcucagaaagccg...................................................................  3      1  Mm3
....................uuuugauuguugcucagaaagccg...................................................................  2263   0  Mm3
....................uuuugauuguugcucaAaaagccg...................................................................  3      1  Mm3
....................uuuugauuguugcucagaGagccg...................................................................  1      1  Mm3
....................uuuugauuguugcucagGaagccg...................................................................  8      1  Mm3
....................uuuugauuguugcucagaaagccA...................................................................  718    1  Mm3
....................uuuugauuguugcucaCaaagccg...................................................................  10     1  Mm3
....................uuuugauuguuAcucagaaagccg...................................................................  1      1  Mm3
....................uuuugauuguugUucagaaagccg...................................................................  1      1  Mm3
....................uuuugauuguugcucaUaaagccg...................................................................  4      1  Mm3
....................Nuuugauuguugcucagaaagccg...................................................................  2      1  Mm3
....................uuuugauuguCgcucagaaagccg...................................................................  1      1  Mm3
....................uuuugauuguGgcucagaaagccg...................................................................  3      1  Mm3
....................uuuugauuguugcucagaaGgccg...................................................................  2      1  Mm3
....................uuuugauuUuugcucagaaagccg...................................................................  1      1  Mm3
....................uuuugauuguugcucagCaagccg...................................................................  18     1  Mm3
....................uuuugauuguugcucagaaagccC...................................................................  197    1  Mm3
....................uuuugauuguAgcucagaaagccg...................................................................  8      1  Mm3
....................Guuugauuguugcucagaaagccg...................................................................  1      1  Mm3
....................uuuugauuguugcucagaaagccAu..................................................................  1      1  Mm3
....................uuuugauuguugcucagaaagccCu..................................................................  5      1  Mm3
....................uuuugauuguugcucagaaagccUu..................................................................  193    1  Mm3
.....................uuugauuguugcucagaaagc.....................................................................  3      0  Mm3
```

```
                    Mature                           Star
caaagagucguuuguccguguuuugauuguugcucagaaagccguccauuuaagggccuucgaguagcaaucaaauucagaggggaacgacgcuucaacauaccugauuac
.....................uuugauuguugcucagaaagcc....................................................................   53     0    Mm3
.....................uuugauuguugcucagCaagcc....................................................................   1      1    Mm3
.....................uuugauuguugcucagUaagcc....................................................................   1      1    Mm3
.....................uuugauuguugcucagaGagcc....................................................................   2      1    Mm3
.....................uuugauuguuUcucagaaagcc....................................................................   4      1    Mm3
.....................uuugauuguugcucagaaaAcc....................................................................   1      1    Mm3
.....................uuugaAuguugcucagaaagccg...................................................................   1      1    Mm3
.....................uuugauuguugcucagaaagccU...................................................................   62     1    Mm3
.....................uuugauuguugcucagaaagccA...................................................................   1      1    Mm3
.....................uuugauuguugcucagaaagccg...................................................................   1      0    Mm3
......................uugauuguugcucagaaagcc....................................................................   2      0    Mm3
......................uugauuguugcucagaaagccU...................................................................   4      1    Mm3
.......................ugauuguugcucagaaagccU...................................................................   1      1    Mm3
........................................................ccuucgaguagcaaucaaauu..................................   6      0    Mm3
........................................................ccuucgaguagcaaucaaauuc.................................   8      0    Mm3
........................................................ccuucgaguagcaaucaaauuA.................................   1      1    Mm3
........................................................ccuucgaguagcaaucaaUuuca................................   1      1    Mm3
........................................................ccuucgaguagcaaucaaauuca................................   18     0    Mm3

...................guuugauuguugcucagaaagc......................................................................   1      0    Fm3
...................Cuuugauuguugcucagaaagcc.....................................................................   5      1    Fm3
....................uuuugauuguugcucaga.........................................................................   24     0    Fm3
....................uuuugauuguugcucagU.........................................................................   1      1    Fm3
....................uuuugauuguugcucagaa........................................................................   24     0    Fm3
....................uuuugauuguugcucagaaU.......................................................................   31     1    Fm3
....................uuuugauuguugcucagUaa.......................................................................   1      1    Fm3
....................uuuugauuguugcucagaaG.......................................................................   2      1    Fm3
....................uuuugauuguGgcucagaaa.......................................................................   1      1    Fm3
....................uuuugauuguugcucagCaa.......................................................................   1      1    Fm3
....................uuuugauuguugcucagaaa.......................................................................   105    0    Fm3
....................uuuugauuguuAcucagaaag......................................................................   1      1    Fm3
....................uuuugauuguugcucagaaag......................................................................   879    0    Fm3
....................uuuugauuguugcucagaaaU......................................................................   14     1    Fm3
....................uuuugaCuguugcucagaaag......................................................................   1      1    Fm3
....................uuuugauuguugcucaUaaag......................................................................   1      1    Fm3
....................uuuugauuguugcucagaaUg......................................................................   1      1    Fm3
....................uuuugauuguugUucagaaag......................................................................   1      1    Fm3
....................uuuugauuguugcucGgaaag......................................................................   1      1    Fm3
....................uuuugauuguugcucagaaaA......................................................................   11     1    Fm3
....................uuuugauuguugcucaCaaag......................................................................   3      1    Fm3
....................uuuugauuguugcucagCaag......................................................................   7      1    Fm3
....................uuGugauuguugcucagaaag......................................................................   1      1    Fm3
....................uuuugauuguAgcucagaaag......................................................................   2      1    Fm3
....................uuuugauuguugcucagUaag......................................................................   2      1    Fm3
....................uuuugauuguAgcucagaaagc.....................................................................   6      1    Fm3
....................uuuugauuguugcucagUaagc.....................................................................   3      1    Fm3
....................uuGugauuguugcucagaaagc.....................................................................   2      1    Fm3
....................uuuugCuuguugcucagaaagc.....................................................................   1      1    Fm3
....................uuuugauuguugcucagGaagc.....................................................................   5      1    Fm3
....................uuuugauuguugcucagaaagG.....................................................................   1      1    Fm3
....................uuuugauuguugcucUgaaagc.....................................................................   1      1    Fm3
....................uuuugauuguugcucagaaaAc.....................................................................   1      1    Fm3
....................uuuugauuguugcGcagaaagc.....................................................................   1      1    Fm3
....................uuuCgauuguugcucagaaagc.....................................................................   1      1    Fm3
....................uCuugauuguugcucagaaagc.....................................................................   1      1    Fm3
....................uAuugauuguugcucagaaagc.....................................................................   2      1    Fm3
....................uuuugauuguugcucagaaagA.....................................................................   16     1    Fm3
....................uuuugauuguugcucaUaaagc.....................................................................   5      1    Fm3
....................uuuugauuguugcucagaaagc.....................................................................   1466   0    Fm3
....................uuuugauuguugcucagaaagU.....................................................................   29     1    Fm3
....................uuuGgauuguugcucagaaagc.....................................................................   1      1    Fm3
....................uuuugauuguugcucagaaGgc.....................................................................   2      1    Fm3
....................uuuugauuguugcucaAaaagc.....................................................................   2      1    Fm3
....................uuuugauuguugcucaCaaagc.....................................................................   5      1    Fm3
....................uuuugauuguugcucagCaagc.....................................................................   4      1    Fm3
....................uuuugauuguugcCcagaaagc.....................................................................   1      1    Fm3
....................uuuugauuguGgcucagaaagc.....................................................................   1      1    Fm3
....................uuuugauuguugcucagaUagcc....................................................................   1      1    Fm3
....................uuuugauuguugcucagaaaUcc....................................................................   1      1    Fm3
....................uuuugauuguugcucagaaagcc....................................................................   7431   0    Fm3
```

Mature **Star**

caaagagucguuuguccguguuuugauuguugcucagaaagccguccauuuaagggccuucgaguagcaaucaaauucagaggggaacgacgcuucaacauaccugauuac			
....................uuugUuuguugcucagaaagcc..	3	1	Fm3
....................uuuugauuCuugcucagaaagcc..	1	1	Fm3
....................uuuugauugAugcucagaaagcc..	1	1	Fm3
....................uuuugauuguugcucGgaaagcc..	1	1	Fm3
....................uuuugauuguugcucagGaagcc..	28	1	Fm3
....................uuuugauuguugcucagaaagcA..	36	1	Fm3
....................uuuugauuguugcucaAaaagcc..	20	1	Fm3
....................uuuugauCguugcucagaaagcc..	1	1	Fm3
....................uuuugauuguugcuUagaaagcc..	2	1	Fm3
....................Cuuugauuguugcucagaaagcc..	7	1	Fm3
....................uuuugGuuguugcucagaaagcc..	2	1	Fm3
....................uuuugauugCugcucagaaagcc..	2	1	Fm3
....................uuuugauuguugcCcagaaagcc..	2	1	Fm3
....................uuuugauuguugcucagUaagcc..	22	1	Fm3
....................uuuugauuAuugcucagaaagcc..	2	1	Fm3
....................uuuuCauuguugcucagaaagcc..	1	1	Fm3
....................uuuugauuguugcucagCaagcc..	27	1	Fm3
....................uuuugauuguugcucUgaaagcc..	2	1	Fm3
....................uuuugauuUuugcucagaaagcc..	4	1	Fm3
....................uuuugauuguCgcucagaaagcc..	5	1	Fm3
....................uuuugauuguugcAcagaaagcc..	3	1	Fm3
....................uuuugauuguugcucagaaagcG..	1	1	Fm3
....................Auuugauuguugcucagaaagcc..	6	1	Fm3
....................Nuuugauuguugcucagaaagcc..	6	1	Fm3
....................uuAugauuguugcucagaaagcc..	2	1	Fm3
....................uuuugauuguugcucagaaagcU..	58	1	Fm3
....................uuuuAauuguugcucagaaagcc..	1	1	Fm3
....................uuuugauuguugcucaUaaagcc..	18	1	Fm3
....................uuGugauuguugcucagaaagcc..	3	1	Fm3
....................uuuugauuguugcucagaaagUc..	1	1	Fm3
....................uuuugauuguugcucagaaaCcc..	2	1	Fm3
....................uuuugNuuguugcucagaaagcc..	2	1	Fm3
....................uuuugauuguugcucagaaCgcc..	1	1	Fm3
....................uuuugauuguugcucagaaUgcc..	1	1	Fm3
....................uCuugauuguugcucagaaagcc..	6	1	Fm3
....................uuuugauuguGgcucagaaagcc..	14	1	Fm3
....................uuuugauuguAgcucagaaagcc..	6	1	Fm3
....................uuuugauuguugcucagaaaAcc..	1	1	Fm3
....................uAuugauuguugcucagaaagcc..	1	1	Fm3
....................uuuAgauuguugcucagaaagcc..	4	1	Fm3
....................uuuugaCuguugcucagaaagcc..	1	1	Fm3
....................uuuugauuguugcucaCaaagcc..	22	1	Fm3
....................uuuugauuguugcuAagaaagcc..	1	1	Fm3
....................uGuugauuguugcucagaaagcc..	1	1	Fm3
....................uuuugauuguugcucagaaGgcc..	10	1	Fm3
....................Guuugauuguugcucagaaagcc..	4	1	Fm3
....................uuCugauuguugcucagaaagcc..	1	1	Fm3
....................uuuugauuguugcucagaaagccU...	8195	1	Fm3
....................uuuugauuguCgcucagaaagccg...	1	1	Fm3
....................uuuugauCguugcucagaaagccg...	1	1	Fm3
....................uuuugauugAugcucagaaagccg...	1	1	Fm3
....................Guuugauuguugcucagaaagccg...	1	1	Fm3
....................uuuugauuguugcucagaaagccA...	15	1	Fm3
....................uuuugauuguugcucagaaagccC...	3	1	Fm3
....................uuuugauuguugcucagaaGgccg...	1	1	Fm3
....................uuuugauAguugcucagaaagccg...	1	1	Fm3
....................uuuugauuguugcucagaaagccg...	8	0	Fm3
....................uuuugaGuguugcucagaaagccg...	1	1	Fm3
....................uuuugauuguugcucagaaagccUu..	4	1	Fm3
.....................uuugauuguugcucagaaag..	1	0	Fm3
.....................uuugauuguugcucagaaagc...	1	0	Fm3
.....................uuugauuguugcucagaaagcc..	9	0	Fm3
.....................uuugauuguugcucagaaagccU...	11	1	Fm3
......................uugauuguugcucagaaag..	5	0	Fm3
......................uugauuguugcucagaaagc...	3	0	Fm3
......................uugauuguugcucagaaagcc..	5	0	Fm3
......................uugauuguugcucagaaagccU...	2	1	Fm3
.......................ugauuguugcucagaaagcc..	3	0	Fm3
.........................auuguugcucagaaagccA...	1	1	Fm3
..........................uuguugcucagaaagccU...	2	1	Fm3

Mature Star

```
caaagagucguuuguccguguuuugauuguugcucagaaagccguccauuuaagggccuucgaguagcaaucaaauucagaggggaacgacgcuucaacauaccugauuac
........................................................ccuucgaguagcaaucaaau...................................    3    0    Fm3
........................................................ccuucgaguagcaaucaaauu..................................    11   0    Fm3
........................................................ccuucgaguagcaaucaaauuc.................................    9    0    Fm3
........................................................ccuucgaguagcaaucaaauuca................................    14   0    Fm3
........................................................ccuucgaguagcaaucaaauucaU...............................    1    1    Fm3
.........................................................cuucgaguagcaaucaUauucag...............................    1    1    Fm3
.........................................................cuucgaguagcaaucaaauucag...............................    4    0    Fm3
```
